# Two triterpenoids from *Rubus fraxinifolius* leaves and their tyrosinase and elastase inhibitory activities

**DOI:** 10.1038/s41598-021-99970-x

**Published:** 2021-10-14

**Authors:** Yesi Desmiaty, Muhammad Hanafi, Fadlina Chany Saputri, Berna Elya, Eko Aditya Rifai, Rezi Riadhi Syahdi

**Affiliations:** 1 Faculty of Pharmacy, Universitas Indonesia, Depok, Indonesia; 2 Faculty of Pharmacy, Pancasila University, Jakarta, Indonesia; 3 Research Centre for Chemistry, Indonesian Institute of Sciences, Jakarta, Indonesia

**Keywords:** Chemical biology, Drug discovery, Plant sciences

## Abstract

Numerous therapeutic compounds have been isolated from naturally abundant organic resources, which may offer economical and sustainable sources of compounds with safe and efficacious biological activities. In the cosmetics industry, natural compounds with anti-aging activities are eagerly sought. Thus, we prepared various extracts from *Rubus fraxinifolius* leaves and used enzyme inhibition assays to isolate compounds with protective effects against skin aging. Two triterpenoids were isolated from *Rubus fraxinifolius* Poir. leaves. The structures were characterized by spectroscopic analyses (LC-ESI-MS, 1D/2D NMR) and comparison to reported data. Compound **1** and **2** were determined as 2,3-O-ethyleneglycol, 19-hydroxyurs-12-en-23,28-dioic acid and 2,3-O-propanediol,19-hydroxyurs-12-en-28-oic acid. Methanol extract and isolates were assessed for their inhibitory effects on elastase and tyrosinase. Compounds **1** and **2** inhibited elastase with IC_50_ 122.199 µg/mL and 98.22 µg/mL, and also inhibited tyrosinase with IC_50_ 207.79 µg/mL and 221.51 µg/mL, respectively. The molecular docking proved that both compounds have affinities toward the enzymes.

## Introduction

Although challenging to achieve, natural anti-aging substances that are safe for humans can be economically and sustainably harvested from abundant natural resources. About 700 species of *Rubus* (Rosaceae) are distributed globally, even though few species are found in the tropics. This genus containing nutrients (sugar, vitamins, etc.), secondary metabolites (triterpenoids, flavonoids, polyphenols, etc.) and have many anti-aging activities (antioxidant, ant elastase, ant tyrosinase, anti-collagenase, anti-UV, anti-inflammatory, wound healing, etc.)^1,2^. In addition, *Rubus* species also reported containing diverse triterpenes with various biological activities^3–7^.

In Indonesia, *R. fraxinifolius* Poir. (Rosaceae) is distributed in Java, Borneo, etc., and popularly known as ‘arben.’ Moreover, some local farmers harvest these plants, and the berry fruit is usually consumed either fresh or frozen. Our previous study demonstrated that *R. fraxinifolius* stem extract has the activity to inhibit elastase, tyrosinase, and as an antioxidant^8^. Some other investigations also showed that the leaf and fruit of *R. fraxinifolius* have a potent antioxidant activity^9,10^. However, the study of *R. fraxinifolius* has been little researched, and no studies of chemical components have been reported.

Elastase is a serine protease enzyme, which has a crucial role in skin wrinkling or sagging through the degradation of dermal elastic fiber (elastin) and causes loss of skin elasticity. One of these is skin fibroblast-derived elastase. Thus, as moderators of elastin fiber degradation that causes skin aging, elastase inhibitors have attracted attention as agents for cosmetic preparations^11,12^. Melanogenesis is mediated by tyrosinase and regulates melanin biosynthesis through a two-stage reaction. In the initial step, _L_-tyrosine is hydroxylated to _L_-3,4-dihydroxyphenylalanine (L-DOPA). In the second step, L-DOPA is oxidized to the corresponding O-quinone. Therefore, natural compounds with tyrosinase inhibitory activity are commonly used in cosmetics that inhibit hyperpigmentation with melanin and hence favor skin whitening^13^. The elastase and tyrosinase enzyme inhibitors could be developed as skin whitening, anti-aging, or anti-wrinkle agents to treat dermatological disorders^14^.

This study isolated ursane triterpenoids from *R. fraxinifolius* leaves and elucidated their structures in spectroscopic analyses using electrospray ionization mass spectroscopy (ESI-MS) and ^1^H NMR, ^13^C NMR, and 2D NMR (DEPT, HSQC, HMQC, and HMBC). Subsequently, we performed assays of elastase and tyrosinase inhibitory activities of crude extracts, fractions, and isolated compounds.

The activity of two selected compounds was also observed through in silico method in this research. A molecular docking approach was performed with DockThor^15,16^. The macromolecules used in this research were obtained from protein data bank (RCSB PDB, 2000) with identity 2y9x and 3hgp for tyrosinase and elastase, respectively^17,18^. The macromolecules were optimized in UCSF Chimera and the molecular docking results were observed with PyMOL^19,20^.

## Results and discussion

**Figure 1:**
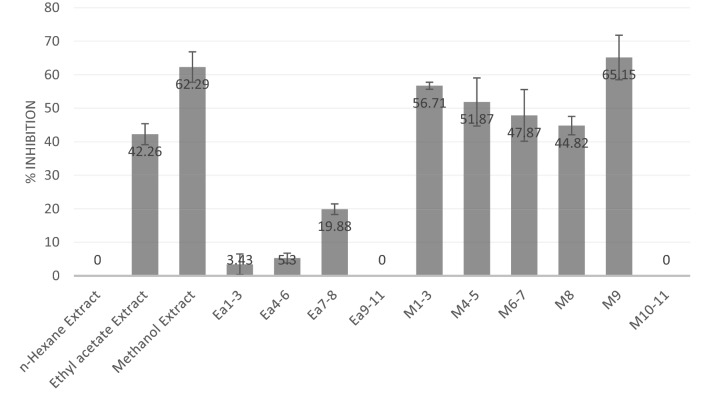
Elastase inhibition by *R. fraxinifolius* leaf extracts and fractions.

The ethyl acetate and methanolic extracts of *R. fraxinifolius* leaves showed potential as elastase inhibitors with percent inhibitory > 40% in 100 µg/mL, whereas n-hexane extract had no activity (Fig. 1). Some Rubus also showed elastase inhibitory activity such *R. sanctus* (% inhibition 14.68–49.20 in 100 µg/mL), R. compactus, R. robustus, etc.^2,21^. Hence, we fractionated the active extracts using vacuum liquid chromatography/VLC and collected 11 fractions from each. Fractions from ethyl acetate extract showed weak elastase inhibitory activities (< 20% in 100 µg/mL), but some methanol fractions showed potential activity in these assays. We choose methanol fraction 8 (M8) for further isolation because it had the largest yield (57%) and also have elastase inhibitory activity (44.82%). M8 was further partitioned and purified over silica gel and through a Sephadex column, and two amorphous powders were produced.

Isolates were identified and characterized using liquid chromatography-mass spectroscopy (LCMS), ^1^H and ^13^C NMR, DEPT, heteronuclear single quantum coherence (HSQC), heteronuclear multiple quantum correlation (HMQC), and heteronuclear multiple bond correlation (HMBC). Table 1, showed the NMR spectral data of compounds.

**Table 1:** ^1^H and ^13^C NMR spectra for compounds **1** and **2**.

		Compound **1** (500 MHz, in CD_3_OD)		Compound **2** (500 MHz, in CD_3_OD)		Tormentic acid^22^ (400 MHz, in CD_3_OD)	
		δ ^13^C	δ ^1^H	δ ^13^C	δ ^1^H	δ ^13^C	δ ^1^H
1	CH_2_	46.39	1.92	39.16	1.7	48.0	2.04
2	CH	68.53	3.85m	78.49	3.35m	68.6	3.62 ddd
3	CH	78.18	3.47 (d,9.5 Hz)	86.09	3.03 (d,10 Hz)	83.8	2.91(d,9.8 Hz)
4	C	41.34	–	43.17	–	41.1	–
5	CH	46.93	1.72	57.15	0.917	55.4	1.04
6	CH_2_	18.40	1.53; 1.28	19.34	1.59; 1.25	19.1	1.59
7	CH_2_	32.6	1.61 (m)	33.65	1.95; 0.903	33.5	1.37/1.64
8	C	39.69	–	44.22	–	40.6	–
9	CH	47.50	1.43	47.96	1.43	47.9	1.82
10	C	37.49	–	42.7	–	39.9	–
11	CH_2_	23.65	2.04; 1.79	23.87	2.04	24.2	2.04
12	CH	127.23	5.3 (t,7)	129.36	5.37	128.2	5.28 (t,3.2)
13	C	139.49	–	140.24	–	139.5	–
14	C	48.39	–	42.84	–	42.3	–
15	CH_2_	28.49	1.83; 1.13	29.66	1.0, 1.76	29.3	1.02, 1.85
16	CH_2_	25.59	1.61;1.36	34.5	1.28; 1,51	26.8	1.75, 1.28
17	C	49.83	–	48.34	–	48.6	–
18	CH	54.20	2.6s	55.15	2.42s	54.4	2.50s
19	C	72.70	–	73.68	–	72.6	–
20	CH	41.73	1.68	41.18	1.68	42.2	1.37
21	CH_2_	26.28	1.53;1.28	27.38	1.53;1.28	26.1	1.54, 2.59
22	CH_2_	38.07	1.77; 2.02	26.69	2.55; 1.51	37.8	1.75
23	C	178.96	–	23.86	1.22s	29.5	0.9s
24	CH_3_	22.63	1.89s	17.46	0.76s	16.8	1.01s
25	CH_3_	17.54	1.02s	17.46	0.97s	17.1	1.08s
26	CH_3_	17.86	0.82s	13.96	0.69s	17.8	0.82s
27	CH_3_	24.98	1.32s	24.86	1.30s	24.6	1.37s
28	C	183.70	–	182.23	–	182.7	–
29	CH_3_	27.36	1.20s	27.17	1.17s	27.0	1.2s
30	CH_3_	16.82	0.92 (d,6)	16.69	0.91 (d,6)	17.5	0.93 (d 6)
31	CH_2_	61.27	3.62 (d,1.15); 4.05 (d,11.5)	66.54	3.22 (d,1.15); 3.42	–	–
32	CH_2_	63.20	3.52 (d,11.5); 4.06 (d,11.5)	20.05	1.4; 1.6	–	–
33	CH_2_	–	–	66.31	4.02 (d,11.5); 3.36	–	–

Data were measured in CD3OD (500 MHz).

**Figure 2:**
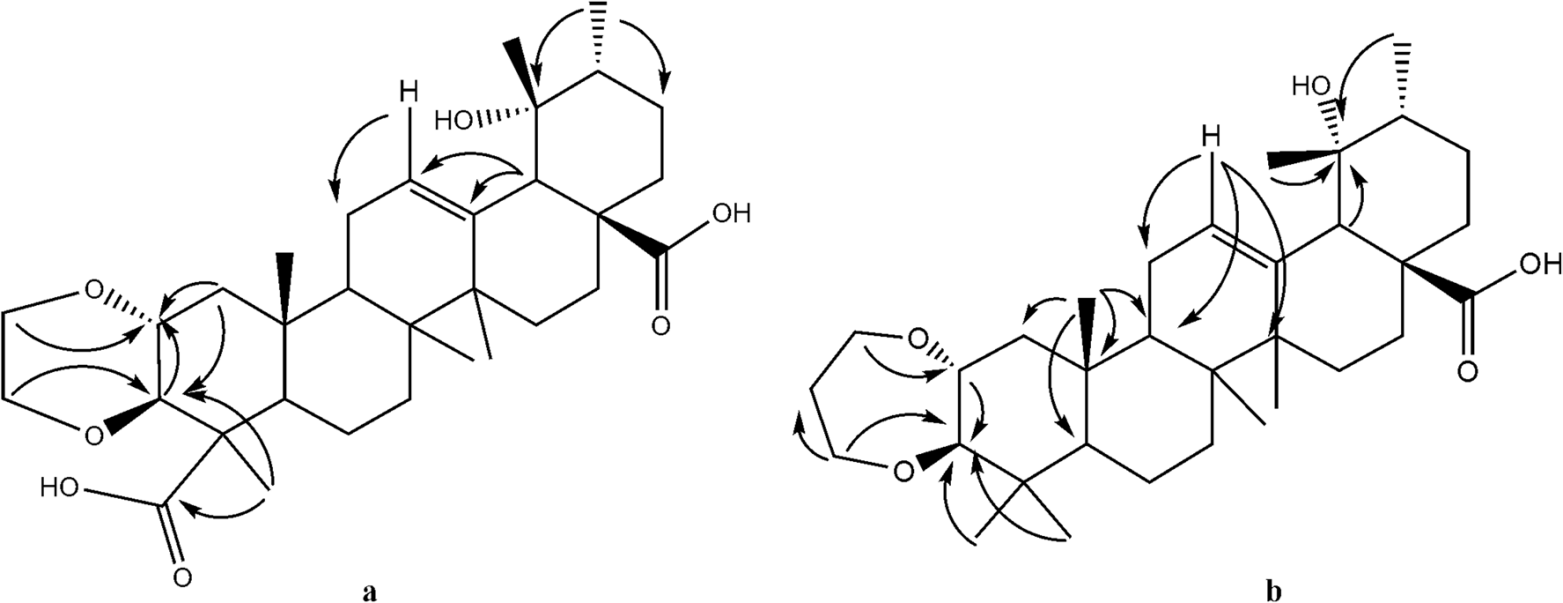
Key HMBC correlations. **a** = compound **1**; **b** = compound **2.**

**Figure 3:**
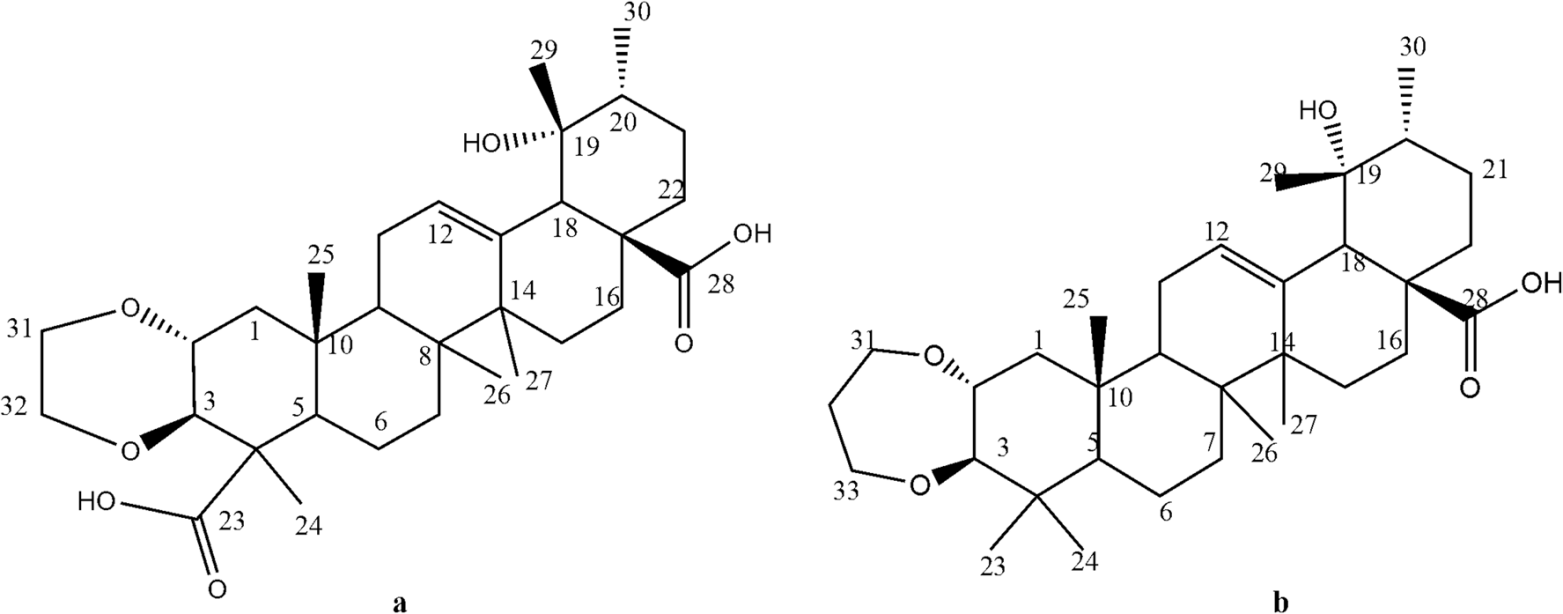
Structures of compounds **1** and **2**. **a** = 2,3-O-ethylene glycol, 19-hydroxyurs-12-en-23,28-dioic acid; **b** = 2,3-O-propanediol, 19-hydroxyurs-12-en-28-oic acid.

Compound **1:** was isolated as an amorphous white powder. LCMS–ESI spectra for this compound displayed a molecular ion peak at m/z 543.32 [M-H]^+^, suggesting the molecular formula C_32_H_48_O_7_ with nine unsaturation equivalents. IR spectra revealed absorption maxima corresponding with hydroxyl (3.343.4 cm^−1^) and olefinic (1.684 cm^−1^) functional groups. Moreover, ^1^H NMR spectra of compound **1** revealed methine proton (CH) a singlet signal at δ_H_ 2.6, a characteristic signal for the H-18 of ursane-type triterpenes with 19-O-substitutions. The triterpene type 19α-hydroxy-ursane also has a typical proton signal in the area around δ_H_ 2.6 ppm. This deshielded signal can differ markedly from other ordinal methylene proton signals, with a characteristic shift due to an olefinic proton (H12) at δ_H_ 5.28 (t, *J* = 6 Hz). Complete and unequivocal ^1^H and ^13^C chemical shift assignments were assisted by HMQC (^13^C × ^1^H) and HMBC (^13^C × ^1^H) spectra. From the HMBC cross peak, the proton at δ_H_ 2.60 was confirmed to be H-16 (Fig. 2a). Other specific characters were found for the five methyl singlets at δ_H_ 1.89, 1.02, 0.82, 1.32, 1.20 (each, H24-27; 29), a methyl doublet at δ_H_ 0.92 (d, *J* = 6 Hz), and the olefinic proton signal at δ_H_ 5.30 (t, *J* = 7 Hz, H-12). The present ^13^C NMR spectra show 32 carbon resonances, and with DEPT and HMQC spectra, displayed two carbonyl carbons (δ C 183.70, C-28 and δ_C_ 178.96, C-23), an olefinic carbon at δ_C_ 127.23, C-12, an olefinic quaternary carbon (δ_C_ 139.49, C-13), an oxygen-bearing quaternary carbon (δ_C_ 72.7, C-19), ten aliphatic methylenes and six methyl carbons. The two olefinic carbons at δ_C_ 127.23 (C-12) and 139.49 (C-13) were characteristic of the ursane-triterpenoid type. Another signal indicated the presence of ethylene glycol (-OCH_2_-CH_2_O-) was showed at signals at δ_H_ 3.62 (d, *J* = 11.5 Hz), 4.05 (d, *J* = 11.5 Hz), 3.52 (d, *J* = 11.5 Hz) and 4.06 (d, J = 11.5 Hz) and supported with δ_C_ 61.27 (t) and 63.20 (t). It was located at C-2 and C-3 based on the presence of long-range correlation in the HMBC spectra. Hence, compound **1** was identified as 2,3-O-ethylene glycol, 19-hydroxyurs-12-en-23,28-dioic acid (Fig. 3a).

Compound **2**: was obtained as a white amorphous powder. MS–ESI m/z 527.33 [M-H]^+^ (calcd. For 528.3815). In LCMS-ESI spectra, a molecular ion peak is present at m/z 527.33 [M-H]^+^, indicating the molecular formula C_33_H_52_O_5_, which required 8 degrees of unsaturation. The IR spectrum contained absorption maxima corresponding with hydroxyl (2,927.8 cm^−1^) and olefinic (1,686 cm^−1^) functional groups. Moreover, ^1^H NMR spectra for compound **2** showed a singlet at δ_H_ 2.42, which is characteristic of the H18 of a ursane-type triterpene with 19-O-substitution. Other specific characteristic spectra included the presence of six methyl singlets at δ_H_ 1.22, 0.76, 0.97, 0.69, 1.30 and 1.17 (each, H-24–28, H-30), a methyl doublet at 0.91 (d, *J* = 7 Hz), and the olefinic proton signal at δ_H_ 5.37 (d, *J* = 7 Hz, H-12). Corresponding ^13^C NMR and DEPT spectra show 33 carbon resonances, and with HSQC and HMQC spectra indicate the presence of a carbonyl carbon (δ_C_ 182.23, C-28), an olefinic carbon (δ_C_ 129.36, C-12), an olefinic quaternary carbon (δ_C_ 140.24, C-13), an oxygen-bearing quaternary carbon (δ_C_ 73.68, C-19), eleven aliphatic methylenes, and seven methyl carbons. These data indicated that compound **2** carries a ursane-triterpenoid skeleton. The other signals are indicated the presence of 1,3-propanediol functional group, at δ_H_ 3.22 (d, *J* = 11.5 Hz), 3.42 (d, *J* = 10.5 Hz), 1.38 (m), 1.51 (m) and 4.02 (d, *J* = 11.5 Hz) and 3.36 (t, *J* = 10.5 Hz). Its functional group has any long-range correlation with C2-C3. Based on these results, compound **2** was thus identified as 2,3-O-Propanediol, 19-hydroxyurs-12-en-28-oic acid (Figs. 2b and 3b). A similar compound is 2,3-O-Isopropylidene tormentic acid, isolated from *Rubus xanthocarpus*.^7^.

In Table 1, all ^1^H and ^13^C NMR spectra were compared with tormentic acid (TA/2α,3α, 19-trihydroxy-12-ursen-28-oic acid)^22^, which is a ursane triterpenoid that has been found in several *Rubus* species^4,6,23–25^. Tormentic acid had strong similarities with compounds **1** and **2**, except that chemical shifts related to C-23 and C31-33 differed. This moiety was also confirmed in DEPT, HMQC, and HMBC experiments (Fig. 2).

The triterpenoid tormentic acid is widely distributed in natural plant foods. It has various bioactivities: hypoglycaemic effects, anti-inflammatory, and anti-atherogenic properties, reduced vascular smooth muscle cell proliferation, antiproliferative activities in renal, prostate, and melanoma cancer cell lines^24,26^. Therefore, because these compounds have the same skeleton, they may have possible appropriate potential activities.

**Figure 4:**
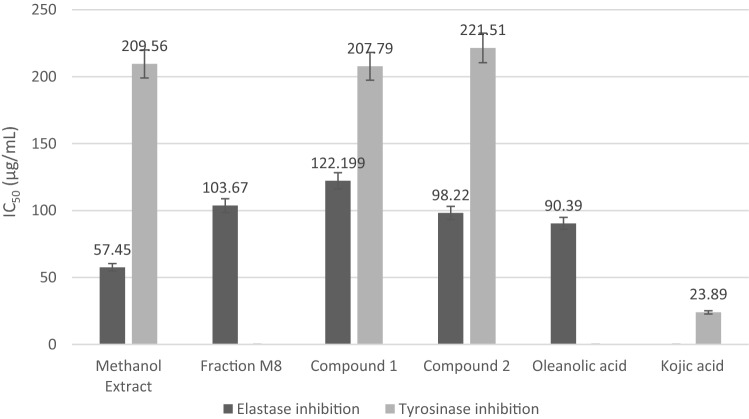
Anti elastase and anti-tyrosinase activities of extracts and isolates from *R. fraxinifolius* leaves.

Figure 4, represents the inhibitory elastase and tyrosinase activity of isolates. The data obtained from in vitro enzyme inhibition assays were expressed as the standard deviation (SD). Compounds **1** and **2** inhibited elastase with IC_50_ 122.199 and 98.22 µg/mL, and also inhibited tyrosinase with IC_50_ 207.79 and 221.51 µg/mL, respectively. Some pentacyclic triterpenoid (ursolic acid and oleanolic acid) reported having elastase inhibition activity^27,28^. The IC_50_ elastase inhibition value of compound **2** is lower than that of compound **1**. Both compounds were less inhibition activity than the positive control oleanolic acid, which had an IC_50_ value of 90.39 µg/mL. In agreement, previous studies showed an IC_50_ for oleanolic acid of 76.5 μg/mL and an IC_50_ value of 31.0 μg/mL for ursolic acid^28^. Previously reported kinetic analyses of pentacyclic triterpenes showed that these compounds competitively and reversibly inhibit neutrophil elastase. In the same study, molecular docking experiments showed that the molecular scaffolding moiety 28-COOH and double bonds in pentacyclic triterpenes are essential for their inhibitory activities^29^.

One of the problems that arise with increasing age is hyperpigmentation. Hence, there is an ongoing search for skin-lightening agents or new depigmenting. Suppression of tyrosinase can act against melanogenesis^30^. As shown in Fig. 4, the methanol extract and compounds **1** and **2** demonstrated moderate activities as tyrosinase inhibitors.

In this research, molecular docking was also used to analyze the binding activities of selected compounds to tyrosinase and elastase as their targets. Crystal structures used were 2Y9X, a crystal structure of tyrosinase from Agaricus bisporus with inhibitor tropolone; and 3HGP, a crystal structure of porcine pancreatic elastase complexed with a potent peptidyl inhibitor FR130180. Both were selected because they were obtained from the same organism used for the in vitro assay of this research. The macromolecules are also bound to their respective inhibitors so that the active state of the enzyme conformations could be obtained. The cocrystals were used as the center of molecular docking target to narrow down the compound binding probabilities so that the scoring process becoming more efficient.

**Figure 5:**
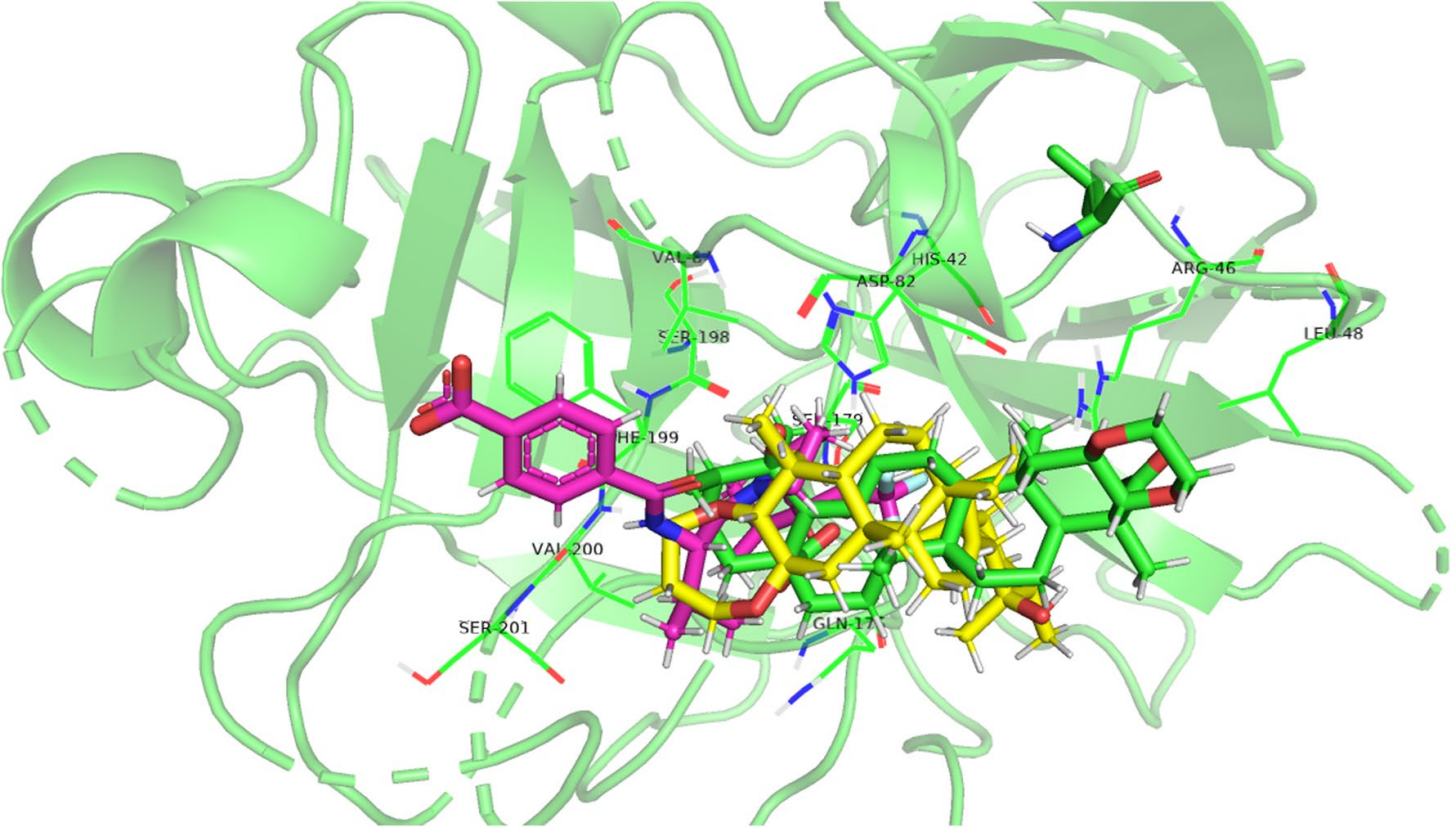
Visualization of elastase binding site using PyMOL. Blue = cocrystal; green = compound 1; yellow = compound 2.

**Figure 6:**
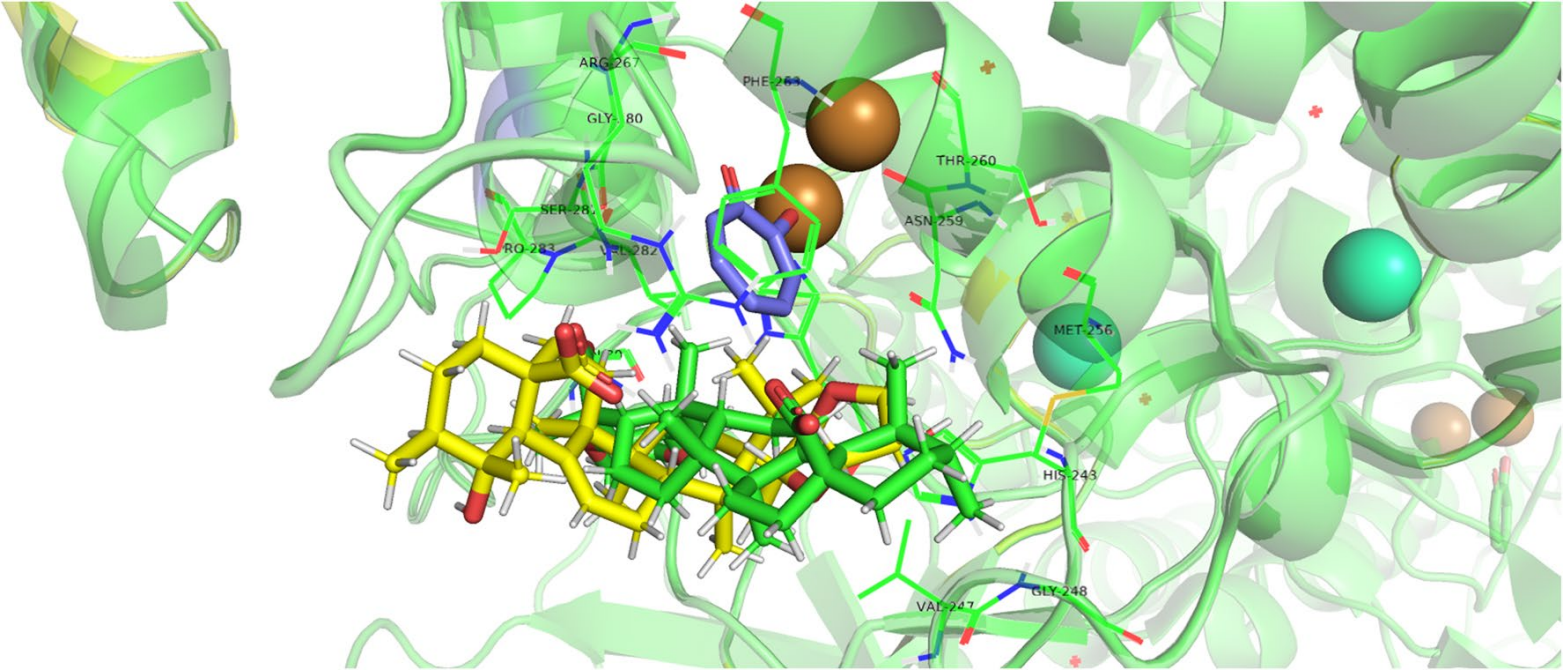
Visualization of tyrosinase binding site using PyMOL. Magenta = cocrystal; green = compound 1; yellow = compound 2.

From the molecular docking, average binding affinities were obtained for both compounds as shown in Figs. 5 and 6. The affinity prediction is used to rank different ligands considering the top energy pose of each compound, a prediction of better energy pose is shown by lower binding affinities scores^16^. The redocking of designated inhibitor tropolone was done with average binding affinities − 7.41 kcal/mol. The average binding affinities of compounds 1 and 2 to tyrosinase were − 7.84 kcal/mol and − 8.37 kcal/mol, respectively (Table 2). Both compounds were predicted to have a better affinity than the inhibitor.

**Table 2:** Binding score of compounds to tyrosinase (PDBID 2Y9X) and elastase (PDBID 3HGP), evaluated using DockThor.

Compound	Average binding score to tyrosinase (kcal/mol)	Average binding score to elastase (kcal/mol)
Compound 1	− 7.84	− 7.58
Compound 2	− 8.37	− 8.06
0TR (2Y9X cocrystal)	− 7.41	
FRW (3HGP cocrystal)		− 7.84

Meanwhile, the average binding affinities of elastase redocking was − 7.84 kcal/mol. The average binding affinities of compounds 1 and 2 to elastase were − 7.58 kcal/mol and -8.06 kcal/mol, respectively. Even though the result showed that compound 1 has lower affinities than the cocrystal, it is slightly different (< 0.5 kcal/mol) compared to the inhibitor used hence the scores may overlap^31^. These scores showed that both compounds were predicted to have affinities toward the enzymes, which is in conjunction with the in vitro assay results.

## Methods

### General experimental procedures

^1^H and ^13^C NMR spectra were recorded at 500 MHz using a JEOL JNM-ECZ500R/S1 instrument. Infrared (IR) spectra were measured with an FTIR, IRPrestige-21, Shimadzu. Other analyses were performed using a Waters UPLC-MS XEVO G2-XS QTof instrument, a VersaMax Microplate reader and a BioTek ELX800 Microplate Reader, pre-coated aluminum sheets TLC-Silica gel 60 GF254 (Merck, Darmstadt, Germany), column chromatography (CC) was performed using Silica gel 60 (Merck, Darmstadt, Germany) with 70–230 mesh for open chromatography and 230–400 mesh for vacuum chromatography.

### Plant materials

*R. fraxinifolius* leaves were collected from plantation area in mount Pangrango, West Java at the height of 4,343 ft, in December 2018. The specimen was identified by a botanist (Dr. Joni Setijo) at the Research Centre for Biology, Indonesian Institute of Sciences, Indonesia, with specimen number 033/IPH.1.01/If.07. The collection of plant material had received permission from farmer and complied with institutional regulations and guidelines.

### Extraction and isolation

Air-dried powdered leaves (2300 g) were extracted using a Soxhlet apparatus with gradient solvent (n-hexane, EtOAc, and MeOH) to provide the respective extracts, then evaporated with a rotary evaporator and vacuum oven. The methanol extract (291 g) and the ethyl acetate extract (65 g) were adsorbed on the silica gel and performed vacuum liquid chromatography/VLC, eluting with a stepwise gradient of EtOAc: MeOH (from 1:0 to 0:1) to produce 11 fractions for each extract (Ea1–Ea11 and M1–M11 and). Similar fractions which had positive reactions with vanillin sulfuric reagents in TLC were combined. Fraction yield: Ea1-3 (2.27 g); Ea4-6 (8.47 g); Ea7-8 (12.9 g); Ea9-11 (22.3 g); and M1-3 (4.89 g); M4-5 (5.1 g); M6-7 (6.02 g); M8 (166.42 g); M9 (10.53 g); M10-11 (2.9 g). All fractions were submitted for inhibitor elastase activity, Fr. M8 gave the greatest yield and had potent activity. Fr. M8 successively further partitioning by CC over silica gel (eluent CH2Cl2/MeOH with a stepwise gradient) and purified using Sephadex LH-20 column (eluting with CHCl3–MeOH 100:10, v/v. Two fractions showed solid nature and were crystallized with chloroform and methanol to get two isolates: compound **1** (18 mg) and **2** (31 mg). The purity of all the isolates was evaluated by two-dimensional TLC and visualize the spot using 5% sulphuric acid in methanol, followed by heating the plates at 110 °C for 5 min.

The crystals were identified and characterized using liquid chromatography-mass spectroscopy (LCMS), ^1^H, and ^13^C distortions enhancement by polarization transfer (DEPT) NMR, heteronuclear single quantum coherence (HSQC), heteronuclear multiple quantum correlation (HMQC), and heteronuclear multiple bond correlation (HMBC).

#### 3-O-ethylene glycol, 19-hydroxyurs-12-en-23,28-dioic acid

White amorphous powder; ^1^H NMR (500 MHz, in CD_3_OD), ^13^C NMR (500 MHz, in CD_3_OD) spectroscopic data see Table 1; MS–ESI m/z 543.32 [M-H]^+^ (calcd for C_32_H_48_O_7_).

#### 3-O-Propanediol, 19-hydroxyurs-12-en-28-oic acid

White amorphous powder; ^1^H NMR (500 MHz, in CD_3_OD), ^13^C NMR (500 MHz, in CD_3_OD) spectroscopic data see Table 1; MS–ESI m/z 527.33 [M-H]^+^ (calcd for C_33_H_52_O_5_).

### Elastase inhibition assay

Elastase inhibition assay was performed as described previously with some modifications^32^. Briefly, in Nunc-96 well microtiter plates, 20-µL aliquots of 0.8-units/mL PPE in Trizma® base buffer (pH 8.0) were mixed with 20-µL samples, and the mixtures were then diluted to 180 µL in Trizma® base buffer. Test extracts were preincubated with enzyme for 15 min, and 20-μL aliquots of the substrate N-succinyl-Ala-Ala-Ala-p-nitroanilide (A3PVN; 2.9 mM) were added and incubated for another 15 min. Positive control and blank wells contained oleanolic acid and water, respectively. Experiments were conducted in triplicate, and inhibition rates were determined according to absorbance at 401 nm using a VersaMax microplate reader.

Percentage inhibition was calculated using the following equation:

$${\text{Elastase inhibition }}\left( \% \right) \, = { 1} - \left[ {\left( {{\text{T}} - {\text{Tb}}} \right)/\left( {{\text{E}} - {\text{Eb}}} \right)} \right] \, \times { 1}00,$$

where E is the absorbance of the enzyme reaction, Eb is the absorbance of the enzyme blank, T is the absorbance of the test sample, and Tb is the absorbance of the test blank. IC_50_ values were determined from a linear graph of percent elastase inhibition against concentration (50, 75, 100,125,150 µg/mL).

### Tyrosinase inhibition assay

Tyrosinase inhibition was determined using the DOPA-chrome formation method as described previously with slight modifications^13^. Briefly, in 96-well plates, 20 μL aliquots of DMSO (control) or test compounds at varying concentrations were mixed with 40 μL aliquots of 30 U/mL mushroom tyrosinase (Sigma Aldrich) and 100-μL of 0.1-M phosphate buffer (pH 6.8). They were preincubated for 10 min at room temperature. Reactions were initiated by adding 4 μL aliquots of 10 mM L-DOPA to each well and incubating at 37 °C for 20 min. Tyrosinase activity was then determined by measuring absorbance at 475 nm. Kojic acid was used as a positive control. Experiments were performed in triplicate. Percentage of tyrosinase inhibition was calculated using the following equation:

$${\text{Tyrosinase inhibition }}\left( \% \right) \, = { 1} - \left[ {\left( {{\text{T}} - {\text{Tb}}} \right)/\left( {{\text{E}} - {\text{Eb}}} \right)} \right] \, \times { 1}00,$$

where E is the absorbance of the enzyme reaction, Eb is the absorbance of the enzyme blank, T is the absorbance of the test sample, and Tb is the absorbance of the test blank. IC_50_ values were determined from a linear graph of percent elastase inhibition against concentration (250, 125, 62.5, 31.25, 15.6 µg/mL).

### Molecular docking

In this research, in silico pharmacological activity was predicted with molecular docking using DockThor. Targeted molecular docking was done by using a cocrystallized ligand as the center of the active site. Structure 2Y9X was used for tyrosinase molecular docking with tropolone, an inhibitor of mushroom tyrosinase, as its cocrystal. The center of the molecular docking site is defined at − 10.032; − 28.769 and − 43.467 as X, Y, Z dimensions respectively. Chain A was separated for use and the Holmium atom was removed from the structure using UCSF Chimera.

The structure of 3HGP, a porcine elastase was also used in this research. FR130180, as the cocrystal was used as the center of the molecular docking site. The coordinates were 12.453, 9.237, and 1.199 for X, Y, Z dimensions, respectively. The binding affinity was analyzed from the ten best conformers of each molecular docking calculation.

## Supplementary Information

Supplementary Information.
