# Supplementary material for: Two triterpenoids from Rubus fraxinifolius leaves and their tyrosinase and elastase inhibitory activities
Source: Sci Rep. 2021 Oct 14;11:20452. doi: 10.1038/s41598-021-99970-x (PMC8516952; doi:10.1038/s41598-021-99970-x)

Two triterpenoids from *Rubus fraxinifolius* leaves and their tyrosinase and elastase inhibitors activities

Yesi Desmiaty[a], Muhammad Hanafi[a,b], Fadlina Chany Saputri[c], and Berna Elya[c*]

[a]*Phytochemistry Department, Faculty of Pharmacy, Pancasila University, Jakarta, Indonesia;*
[b]*Research Centre for Chemistry, Indonesian Institute of Sciences, Jakarta, Indonesia*
[c]*Faculty of Pharmacy, Universitas Indonesia, Depok, Indonesia;*
**Correspondence: berna.elya@farmasi.ui.ac.id; Tel.: +6281314161497*

Supplement 1_NMR and LCMS Compound 1

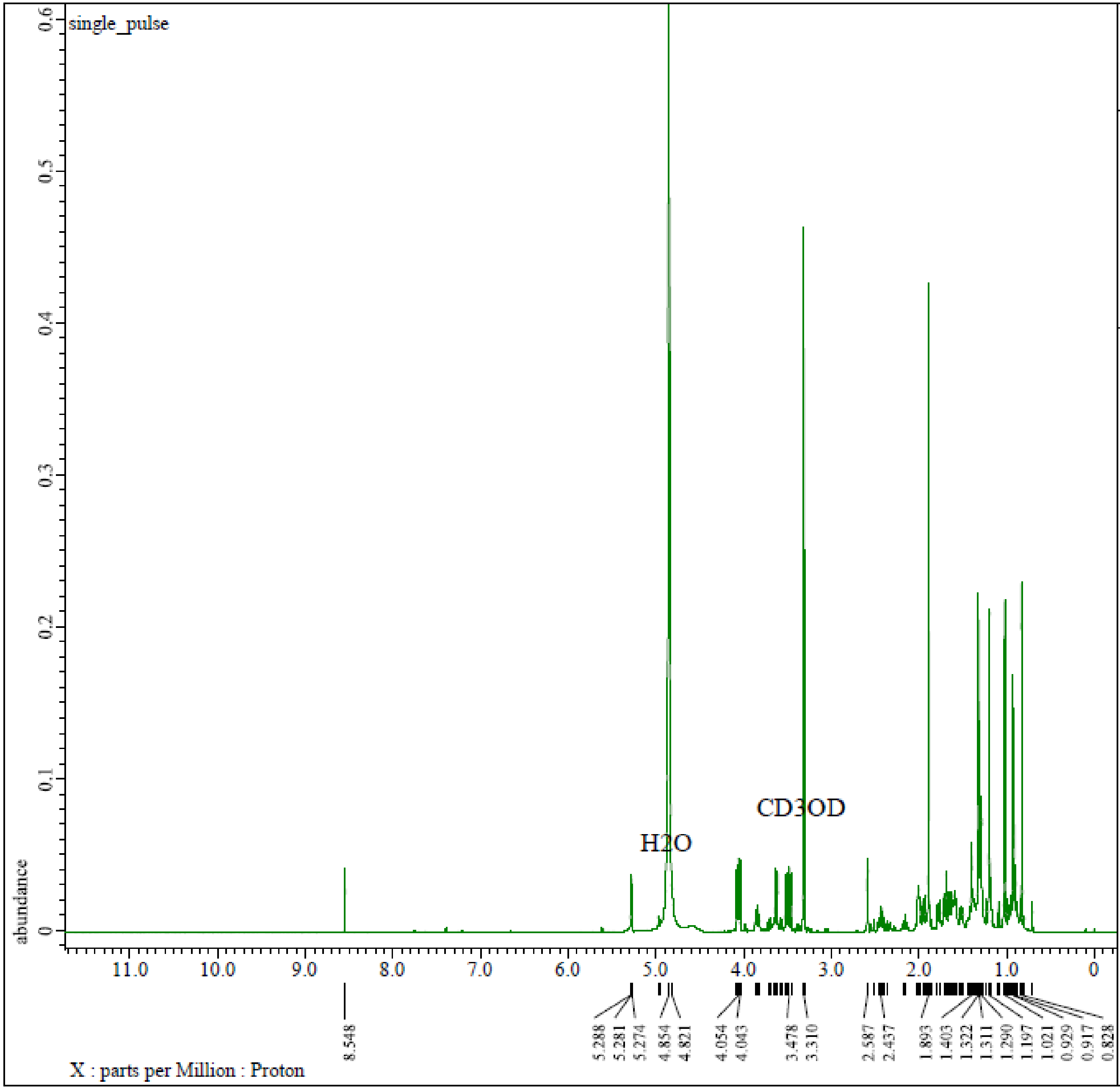


```
---- PROCESSING PARAMETERS ----
sexp( 0.2[Hz], 0.0[s] )
trapezoid( 0[%], 0[%], 80[%], 100[%] )
zerofill( 1 )
fft( 1, TRUE, TRUE )
machinephase
ppm
```

```
Filename              = YESI 3_Proton-1-2.j
Author                = delta
Experiment            = proton.jxp
Sample_Id             = YESI 3
Solvent               = METHANOL-D4
Actual_Start_Time     = 18-JAN-2019 09:39:4
Revision_Time         = 18-JAN-2019 17:23:1

Comment               = single_pulse
Data_Format           = 1D COMPLEX
Dim_Size              = 13107
X_Domain              = Proton
Dim_Title             = Proton
Dim_Units             = [ppm]
Dimensions            = X
Spectrometer          = JNM-ECZ500R/S1

Field_Strength        = 11.7473579[T]  (500[
X_Acq_Duration        = 1.74587904[s]
X_Domain              = Proton
X_Freq                = 500.15991521[MHz]
X_Offset              = 5.0[ppm]
X_Points              = 16384
X_Prescans            = 1
X_Resolution          = 0.57277737[Hz]
X_Sweep               = 9.38438438[kHz]
X_Sweep_Clipped       = 7.50750751[kHz]
Irr_Domain            = Proton
Irr_Freq              = 500.15991521[MHz]
Irr_Offset            = 5.0[ppm]
Tri_Domain            = Proton
Tri_Freq              = 500.15991521[MHz]
Tri_Offset            = 5.0[ppm]
Blanking              = 2[us]
Clipped               = FALSE
Scans                 = 80
Total_Scans           = 80

Relaxation_Delay      = 5[s]
Recvr_Gain            = 46
Temp_Get              = 25.9[dC]
X_90_Width            = 8[us]
X_Acq_Time            = 1.74587904[s]
X_Angle               = 45[deg]
X_Atn                 = 8.9[dB]
X_Pulse               = 4[us]
Irr_Mode              = Off
Tri_Mode              = Off
Dante_Loop            = 500
```

You created this PDF from an application that is not licensed to print to novaPDF printer (http://www.novapdf.com)

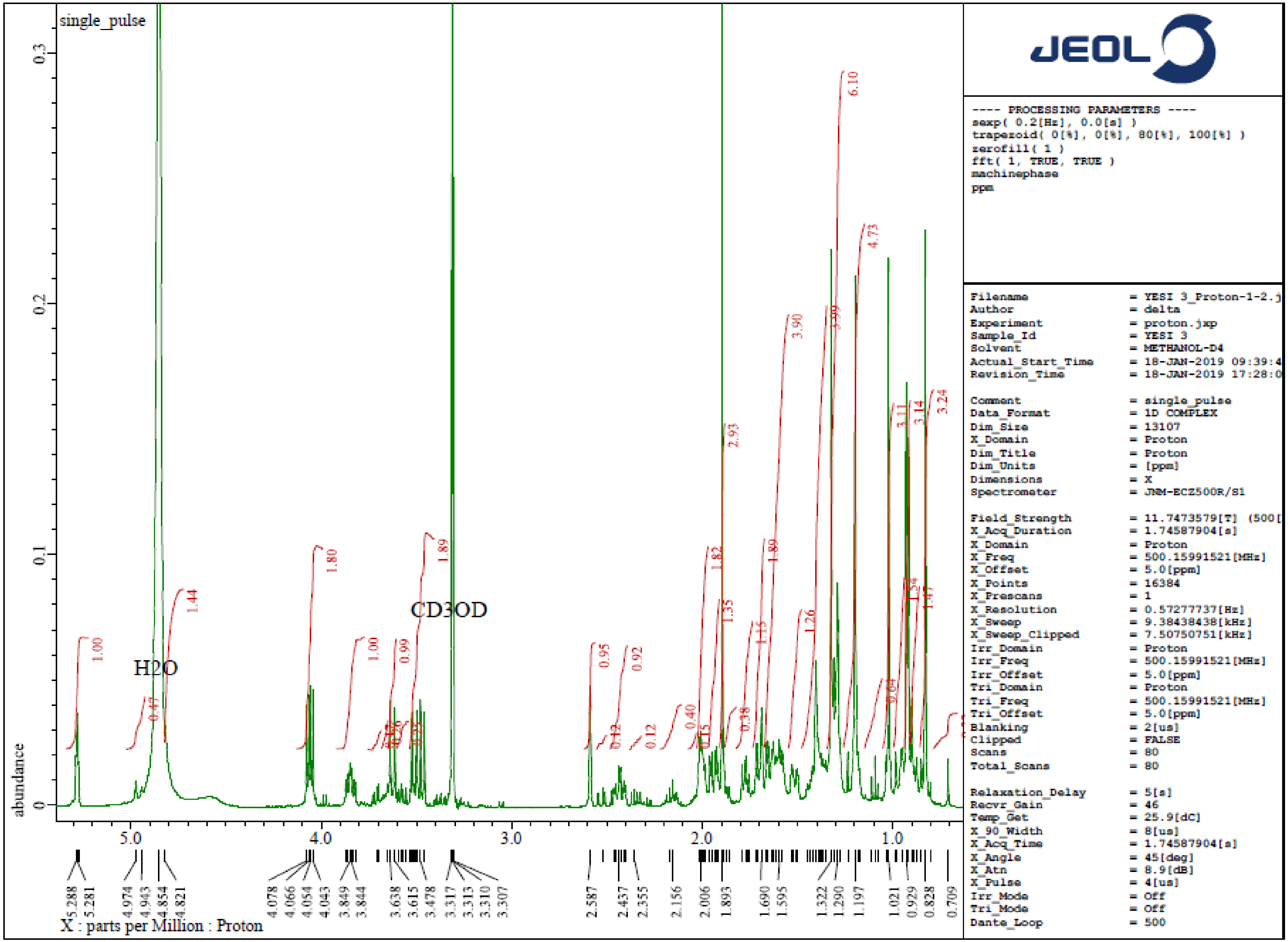

---- PROCESSING PARAMETERS ----
sexp(0.2[Hz], 0.0[s])
trapezoid(0[%], 0[%], 80[%], 100[%])
zerofill(1)
fft(1, TRUE, TRUE)
machinephase
ppm

Filename	= YESI 3_Proton-1-2.j
Author	= delta
Experiment	= proton.jxp
Sample_Id	= YESI 3
Solvent	= METHANOL-D4
Actual_Start_Time	= 18-JAN-2019 09:39:4
Revision_Time	= 18-JAN-2019 17:28:0
Comment	= single_pulse
Data_Format	= 1D COMPLEX
Dim_Size	= 13107
X_Domain	= Proton
Dim_Title	= Proton
Dim_Units	= [ppm]
Dimensions	= X
Spectrometer	= JNM-ECZ500R/S1
Field_Strength	= 11.7473579[T] (500[
X_Acq_Duration	= 1.74587904[s]
X_Domain	= Proton
X_Freq	= 500.15991521[MHz]
X_Offset	= 5.0[ppm]
X_Points	= 16384
X_Prescans	= 1
X_Resolution	= 0.57277737[Hz]
X_Sweep	= 9.38438438[kHz]
X_Sweep_Clipped	= 7.50750751[kHz]
Irr_Domain	= Proton
Irr_Freq	= 500.15991521[MHz]
Irr_Offset	= 5.0[ppm]
Tri_Domain	= Proton
Tri_Freq	= 500.15991521[MHz]
Tri_Offset	= 5.0[ppm]
Blanking	= 2[us]
Clipped	= FALSE
Scans	= 80
Total_Scans	= 80
Relaxation_Delay	= 5[s]
Recvr_Gain	= 46
Temp_Get	= 25.9[dC]
X_90_Width	= 8[us]
X_Acq_Time	= 1.74587904[s]
X_Angle	= 45[deg]
X_Atn	= 8.9[dB]
X_Pulse	= 4[us]
Irr_Mode	= Off
Tri_Mode	= Off
Dante_Loop	= 500

You created this PDF from an application that is not licensed to print to novaPDF printer (http://www.novapdf.com)

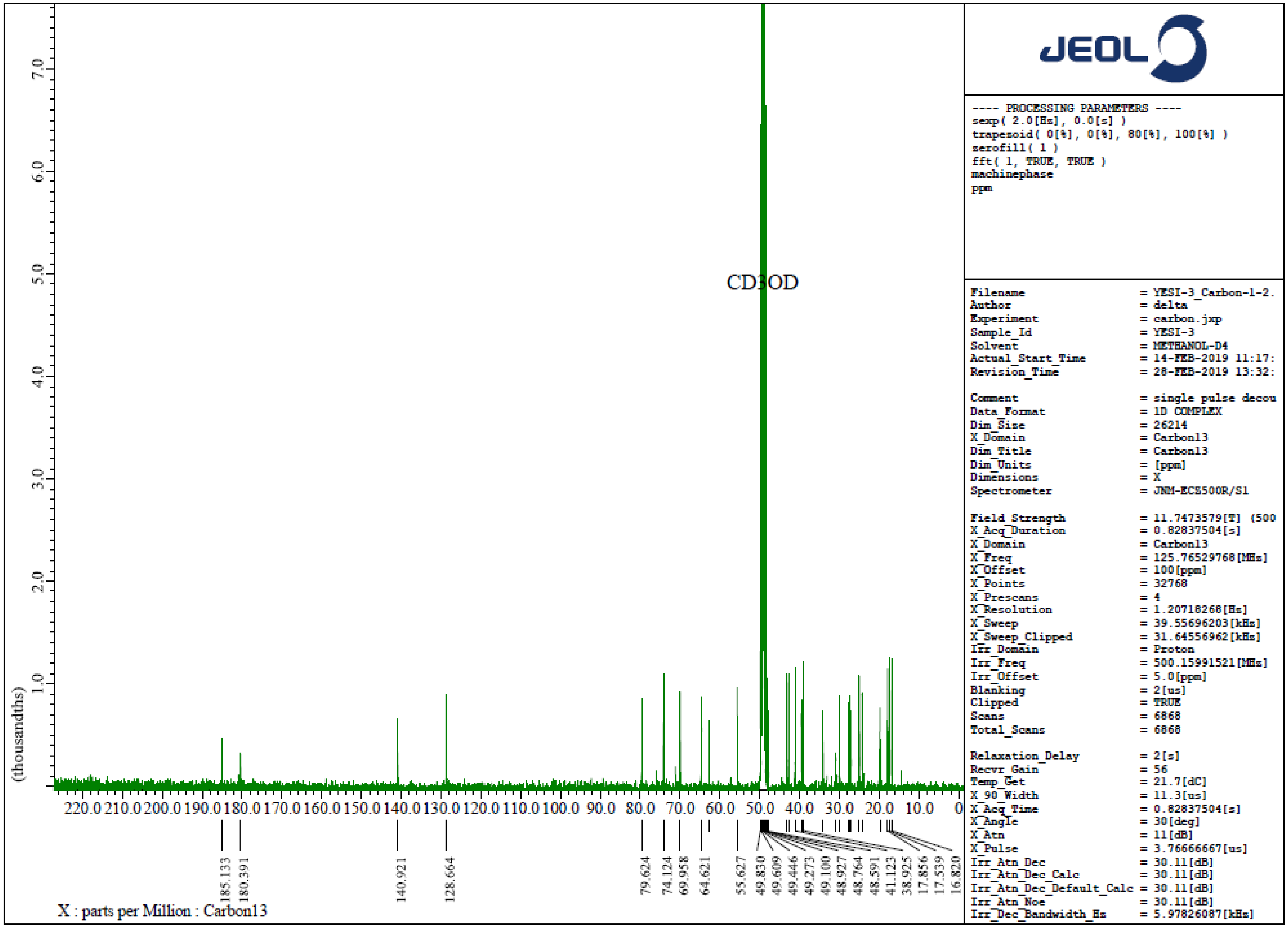

---- PROCESSING PARAMETERS ----
sexp(2.0[Hz], 0.0[s])
trapezoid(0[%], 0[%], 80[%], 100[%])
zerofill(1)
fft(1, TRUE, TRUE)
machinephase
ppm

Filename	= YESI-3_Carbon-1-2.
Author	= delta
Experiment	= carbon.jxp
Sample_Id	= YESI-3
Solvent	= METHANOL-D4
Actual_Start_Time	= 14-FEB-2019 11:17:
Revision_Time	= 28-FEB-2019 13:32:
Comment	= single pulse decou
Data_Format	= 1D COMPLEX
Dim_Size	= 26214
X_Domain	= Carbon13
Dim_Title	= Carbon13
Dim_Units	= [ppm]
Dimensions	= X
Spectrometer	= JNM-ECZ500R/S1
Field_Strength	= 11.7473579[T] (500
X_Acq_Duration	= 0.82837504[s]
X_Domain	= Carbon13
X_Freq	= 125.76529768[MHz]
X_Offset	= 100[ppm]
X_Points	= 32768
X_Prescans	= 4
X_Resolution	= 1.20718268[Hz]
X_Sweep	= 39.55696203[kHz]
X_Sweep_Clipped	= 31.64556962[kHz]
Irr_Domain	= Proton
Irr_Freq	= 500.15991521[MHz]
Irr_Offset	= 5.0[ppm]
Blanking	= 2[us]
Clipped	= TRUE
Scans	= 6868
Total_Scans	= 6868
Relaxation_Delay	= 2[s]
Recvr_Gain	= 56
Temp_Get	= 21.7[dC]
X_90_Width	= 11.3[us]
X_Acq_Time	= 0.82837504[s]
X_Angle	= 30[deg]
X_Atn	= 11[dB]
X_Pulse	= 3.76666667[us]
Irr_Atn_Dec	= 30.11[dB]
Irr_Atn_Dec_Calc	= 30.11[dB]
Irr_Atn_Dec_Default_Calc	= 30.11[dB]
Irr_Atn_Noe	= 30.11[dB]
Irr_Dec_Bandwidth_Hz	= 5.97826087[kHz]

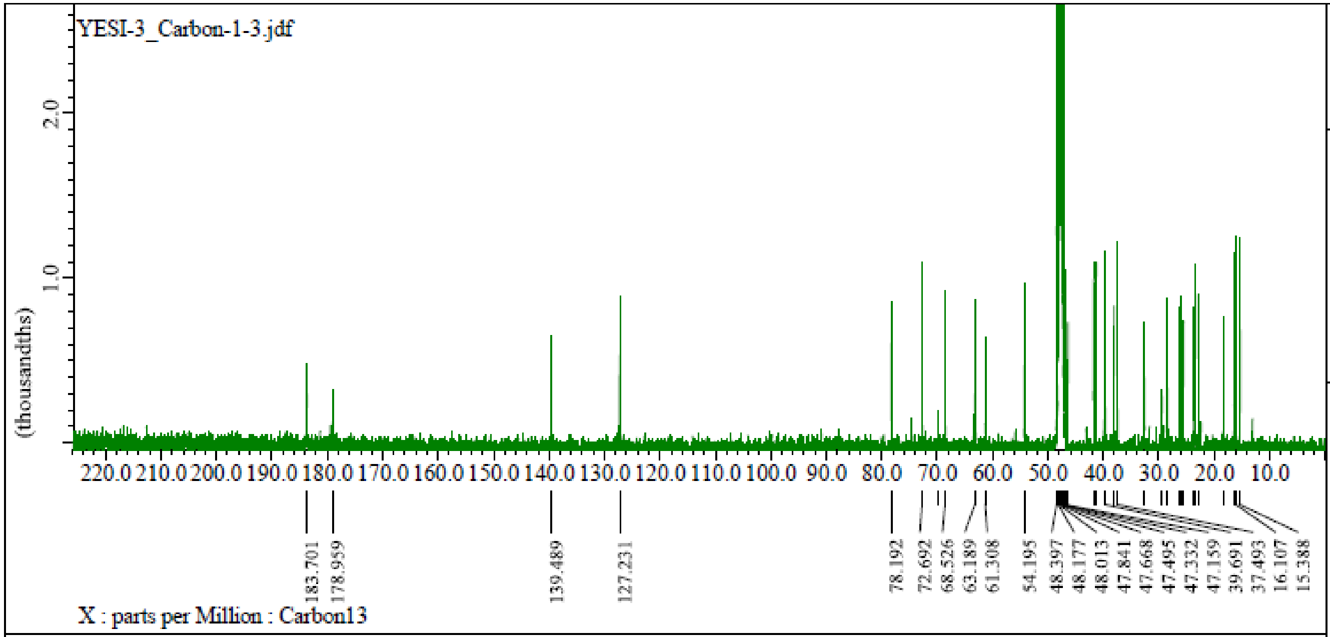

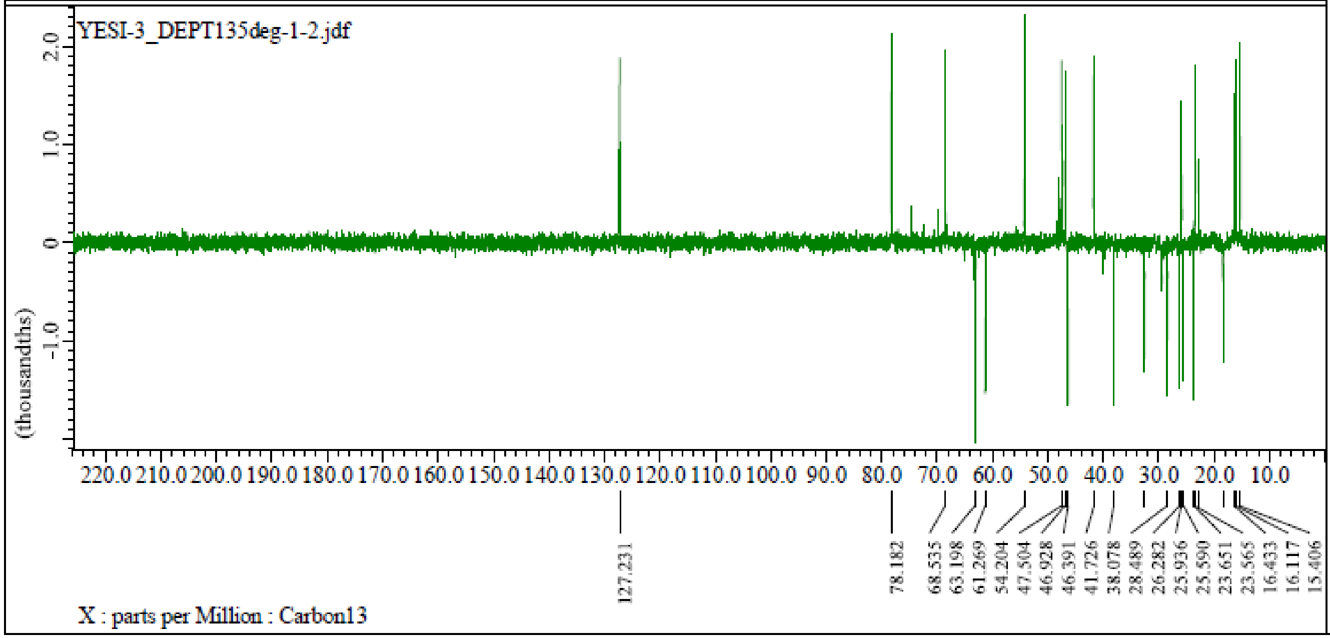


```
---- PROCESSING PARAMETERS ----
sexp( 2.0[Hz], 0.0[s] )
trapezoid( 0[%], 0[%], 80[%], 100[%] )
zerofill( 1 )
fft( 1, TRUE, TRUE )
machinephase
ppm

Filename                  = YESI-3_Carbon-1-3.
Author                    = delta
Experiment                = carbon.jxp
Sample_Id                 = YESI-3
Solvent                   = METHANOL-D4
Actual_Start_Time         = 14-FEB-2019 11:17:
Revision_Time             =  8-MAR-2019 13:47:

Comment                   = single pulse decou
Data_Format               = 1D COMPLEX
Dim_Size                  = 26214
X_Domain                  = Carbon13
Dim_Title                 = Carbon13
Dim_Units                 = [ppm]
Dimensions                = X
Spectrometer              = JNM-ECZ500R/S1

Field_Strength            = 11.7473579[T] (500
X_Acq_Duration            = 0.82837504[s]
X_Domain                  = Carbon13
X_Freq                    = 125.76529768[MHz]
X_Offset                  = 100[ppm]
X_Points                  = 32768
X_Prescans                = 4
X_Resolution              = 1.20718268[Hz]
X_Sweep                   = 39.55696203[kHz]
X_Sweep_Clipped           = 31.64556962[kHz]
Irr_Domain                = Proton
Irr_Freq                  = 500.15991521[MHz]
Irr_Offset                = 5.0[ppm]
Blanking                  = 2[us]
Clipped                   = TRUE
Scans                     = 6868
Total_Scans               = 6868

Relaxation_Delay          = 2[s]
Recvr_Gain                = 56
Temp_Get                  = 21.7[dC]
X_90_Width                = 11.3[us]
X_Acq_Time                = 0.82837504[s]
X_Angle                   = 30[deg]
X_Atn                     = 11[dB]
X_Pulse                   = 3.76666667[us]
Irr_Atn_Dec               = 30.11[dB]
Irr_Atn_Dec_Calc          = 30.11[dB]
Irr_Atn_Dec_Default_Calc  = 30.11[dB]
Irr_Atn_Noe               = 30.11[dB]
Irr_Dec_Bandwidth_Hz      = 5.97826087[kHz]
```

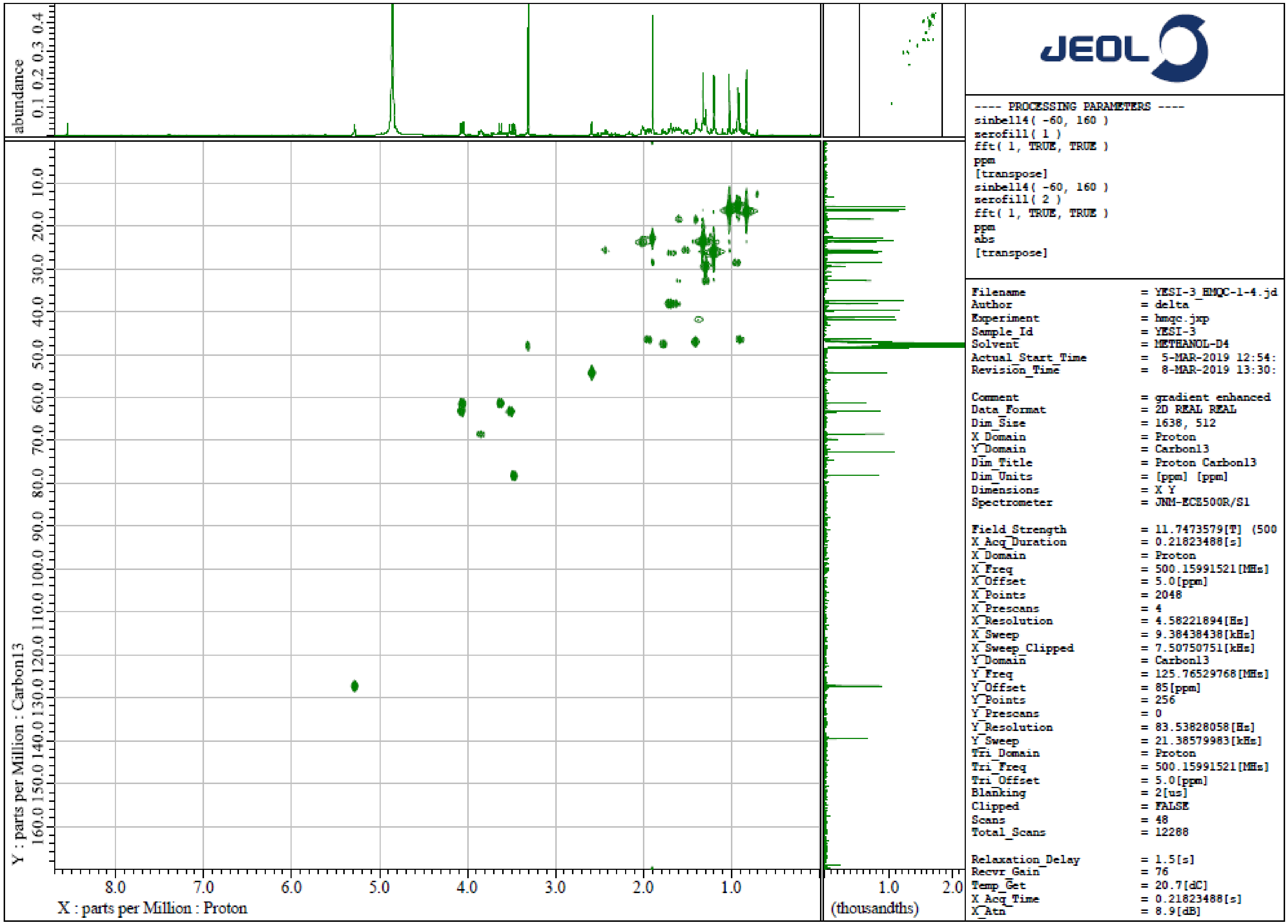

JEOL

```
---- PROCESSING PARAMETERS ----
sinbell4( -60, 160 )
zerofill( 1 )
fft( 1, TRUE, TRUE )
ppm
[transpose]
sinbell4( -60, 160 )
zerofill( 2 )
fft( 1, TRUE, TRUE )
ppm
abs
[transpose]
```

```
Filename                = YESI-3_HMQC-1-4.jd
Author                  = delta
Experiment              = hmqc.jxp
Sample_Id               = YESI-3
Solvent                 = METHANOL-D4
Actual_Start_Time       =  5-MAR-2019 12:54:
Revision_Time           =  8-MAR-2019 13:30:

Comment                 = gradient enhanced
Data_Format             = 2D REAL REAL
Dim_Size                = 1638, 512
X_Domain                = Proton
Y_Domain                = Carbon13
Dim_Title               = Proton Carbon13
Dim_Units               = [ppm] [ppm]
Dimensions              = X Y
Spectrometer            = JNM-ECZ500R/S1

Field_Strength          = 11.7473579[T] (500
X_Acq_Duration          = 0.21823488[s]
X_Domain                = Proton
X_Freq                  = 500.15991521[MHz]
X_Offset                = 5.0[ppm]
X_Points                = 2048
X_Prescans              = 4
X_Resolution            = 4.58221894[Hz]
X_Sweep                 = 9.38438438[kHz]
X_Sweep_Clipped         = 7.50750751[kHz]
Y_Domain                = Carbon13
Y_Freq                  = 125.76529768[MHz]
Y_Offset                = 85[ppm]
Y_Points                = 256
Y_Prescans              = 0
Y_Resolution            = 83.53828058[Hz]
Y_Sweep                 = 21.38579983[kHz]
Tri_Domain              = Proton
Tri_Freq                = 500.15991521[MHz]
Tri_Offset              = 5.0[ppm]
Blanking                = 2[us]
Clipped                 = FALSE
Scans                   = 48
Total_Scans             = 12288

Relaxation_Delay        = 1.5[s]
Recvr_Gain              = 76
Temp_Get                = 20.7[dC]
X_Acq_Time              = 0.21823488[s]
X_Atn                   = 8.9[dB]
```

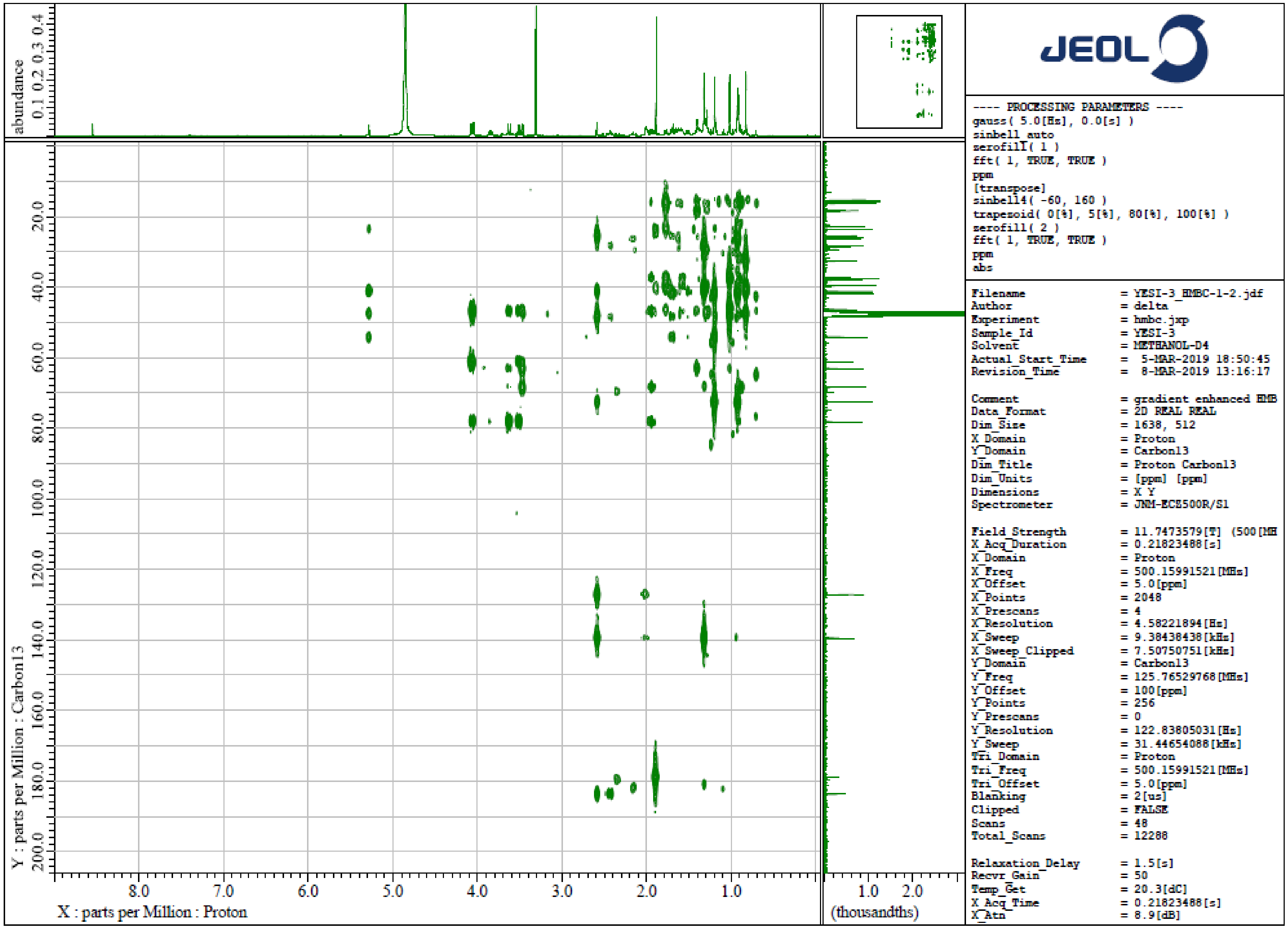


```
---- PROCESSING PARAMETERS ----
gauss( 5.0[Hz], 0.0[s] )
sinbell_auto
zerofill( 1 )
fft( 1, TRUE, TRUE )
ppm
[transpose]
sinbell4( -60, 160 )
trapezoid( 0[%], 5[%], 80[%], 100[%] )
zerofill( 2 )
fft( 1, TRUE, TRUE )
ppm
abs
```

```
Filename              = YESI-3_HMBC-1-2.jdf
Author                = delta
Experiment            = hmbc.jxp
Sample_Id             = YESI-3
Solvent               = METHANOL-D4
Actual_Start_Time     =  5-MAR-2019 18:50:45
Revision_Time         =  8-MAR-2019 13:16:17

Comment               = gradient enhanced HMB
Data_Format           = 2D REAL REAL
Dim_Size              = 1638, 512
X_Domain              = Proton
Y_Domain              = Carbon13
Dim_Title             = Proton Carbon13
Dim_Units             = [ppm] [ppm]
Dimensions            = X Y
Spectrometer          = JNM-ECZ500R/S1

Field_Strength        = 11.7473579[T] (500[MH
X_Acq_Duration        = 0.21823488[s]
X_Domain              = Proton
X_Freq                = 500.15991521[MHz]
X_Offset              = 5.0[ppm]
X_Points              = 2048
X_Prescans            = 4
X_Resolution          = 4.58221894[Hz]
X_Sweep               = 9.38438438[kHz]
X_Sweep_Clipped       = 7.50750751[kHz]
Y_Domain              = Carbon13
Y_Freq                = 125.76529768[MHz]
Y_Offset              = 100[ppm]
Y_Points              = 256
Y_Prescans            = 0
Y_Resolution          = 122.83805031[Hz]
Y_Sweep               = 31.44654088[kHz]
Tri_Domain            = Proton
Tri_Freq              = 500.15991521[MHz]
Tri_Offset            = 5.0[ppm]
Blanking              = 2[us]
Clipped               = FALSE
Scans                 = 48
Total_Scans           = 12288

Relaxation_Delay      = 1.5[s]
Recvr_Gain            = 50
Temp_Get              = 20.3[dC]
X_Acq_Time            = 0.21823488[s]
X_Atn                 = 8.9[dB]
```

Created by: Administrator, UNIFI

Created on: Jul 18, 2019

Item name: Process_Data_Yesi_FFUI

Created time: 09:42:00 SE Asia Standard Time

Item name: BFM
Item description:

Channel name: High energy : Time 7.3470 +/- 0.1023 minutes

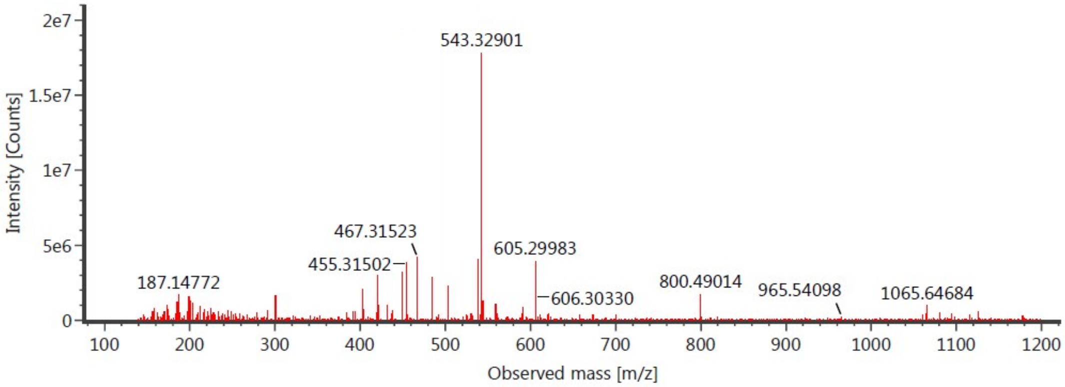

Supplement 2_NMR and LCMS Compound 2

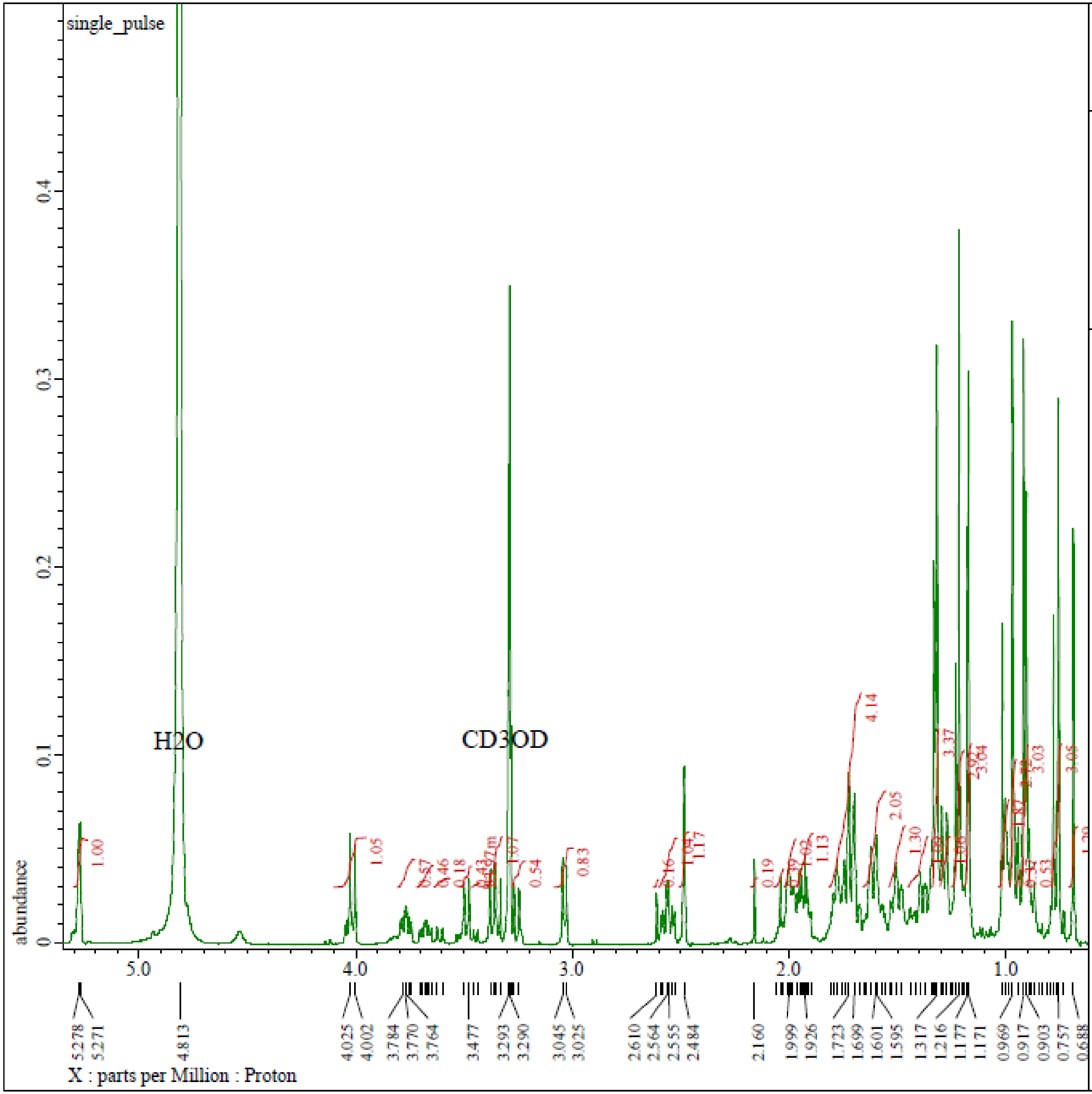


```
---- PROCESSING PARAMETERS ----
sexp( 0.2[Hz], 0.0[s] )
trapezoid( 0[%], 0[%], 80[%], 100[%] )
zerofill( 1 )
fft( 1, TRUE, TRUE )
machinephase
ppm
```

```
Filename            = YESI 5_Proton-1-2.j
Author              = delta
Experiment          = proton.jxp
Sample_Id           = YESI 5
Solvent             = METHANOL-D4
Actual_Start_Time   = 16-JAN-2019 14:22:2
Revision_Time       = 16-JAN-2019 14:48:5

Comment             = single_pulse
Data_Format         = 1D COMPLEX
Dim_Size            = 13107
X_Domain            = Proton
Dim_Title           = Proton
Dim_Units           = [ppm]
Dimensions          = X
Spectrometer        = JNM-ECZ500R/S1

Field_Strength      = 11.7473579[T] (500[
X_Acq_Duration      = 1.74587904[s]
X_Domain            = Proton
X_Freq              = 500.15991521[MHz]
X_Offset            = 7.0[ppm]
X_Points            = 16384
X_Prescans          = 1
X_Resolution        = 0.57277737[Hz]
X_Sweep             = 9.38438438[kHz]
X_Sweep_Clipped     = 7.50750751[kHz]
Irr_Domain          = Proton
Irr_Freq            = 500.15991521[MHz]
Irr_Offset          = 5.0[ppm]
Tri_Domain          = Proton
Tri_Freq            = 500.15991521[MHz]
Tri_Offset          = 5.0[ppm]
Blanking            = 2[us]
Clipped             = FALSE
Scans               = 80
Total_Scans         = 80

Relaxation_Delay    = 5[s]
Recvr_Gain          = 46
Temp_Get            = 27[dC]
X_90_Width          = 8[us]
X_Acq_Time          = 1.74587904[s]
X_Angle             = 45[deg]
X_Atn               = 8.9[dB]
X_Pulse             = 4[us]
Irr_Mode            = Off
Tri_Mode            = Off
Dante_Loop          = 500
```

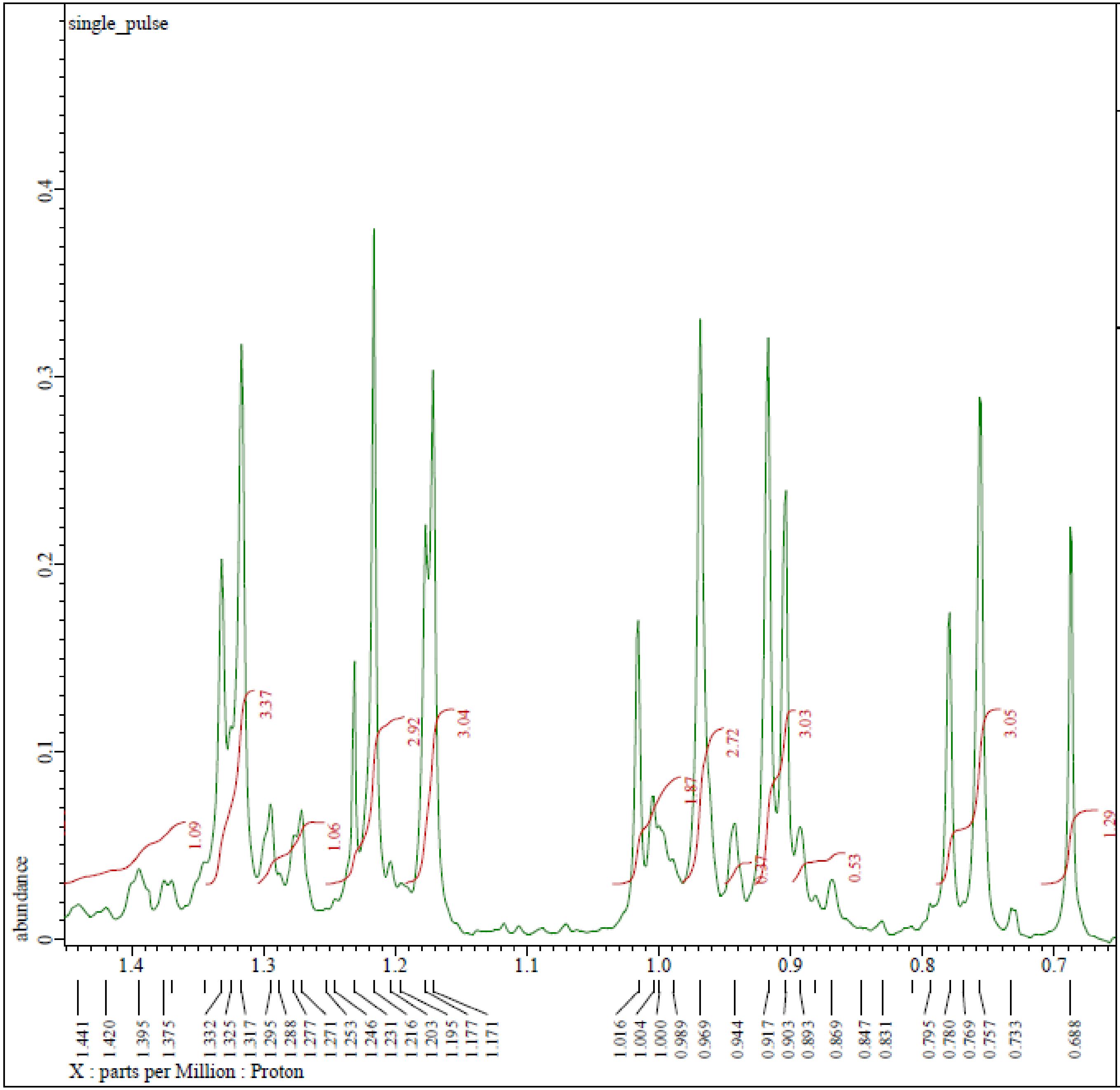

JEOL

```
---- PROCESSING PARAMETERS ----
sexp( 0.2[Hz], 0.0[s] )
trapezoid( 0[%], 0[%], 80[%], 100[%] )
zerofill( 1 )
fft( 1, TRUE, TRUE )
machinephase
ppm
```

```
Filename            = YESI 5_Proton-1-2.j
Author              = delta
Experiment          = proton.jxp
Sample_Id           = YESI 5
Solvent             = METHANOL-D4
Actual_Start_Time   = 16-JAN-2019 14:22:2
Revision_Time       = 16-JAN-2019 14:48:5

Comment             = single_pulse
Data_Format         = 1D COMPLEX
Dim_Size            = 13107
X_Domain            = Proton
Dim_Title           = Proton
Dim_Units           = [ppm]
Dimensions          = X
Spectrometer        = JNM-ECZ500R/S1

Field_Strength      = 11.7473579[T] (500[
X_Acq_Duration      = 1.74587904[s]
X_Domain            = Proton
X_Freq              = 500.15991521[MHz]
X_Offset            = 7.0[ppm]
X_Points            = 16384
X_Prescans          = 1
X_Resolution        = 0.57277737[Hz]
X_Sweep             = 9.38438438[kHz]
X_Sweep_Clipped     = 7.50750751[kHz]
Irr_Domain          = Proton
Irr_Freq            = 500.15991521[MHz]
Irr_Offset          = 5.0[ppm]
Tri_Domain          = Proton
Tri_Freq            = 500.15991521[MHz]
Tri_Offset          = 5.0[ppm]
Blanking            = 2[us]
Clipped             = FALSE
Scans               = 80
Total_Scans         = 80

Relaxation_Delay    = 5[s]
Recvr_Gain          = 46
Temp_Get            = 27[dC]
X_90_Width          = 8[us]
X_Acq_Time          = 1.74587904[s]
X_Angle             = 45[deg]
X_Atn               = 8.9[dB]
X_Pulse             = 4[us]
Irr_Mode            = Off
Tri_Mode            = Off
Dante_Loop          = 500
```

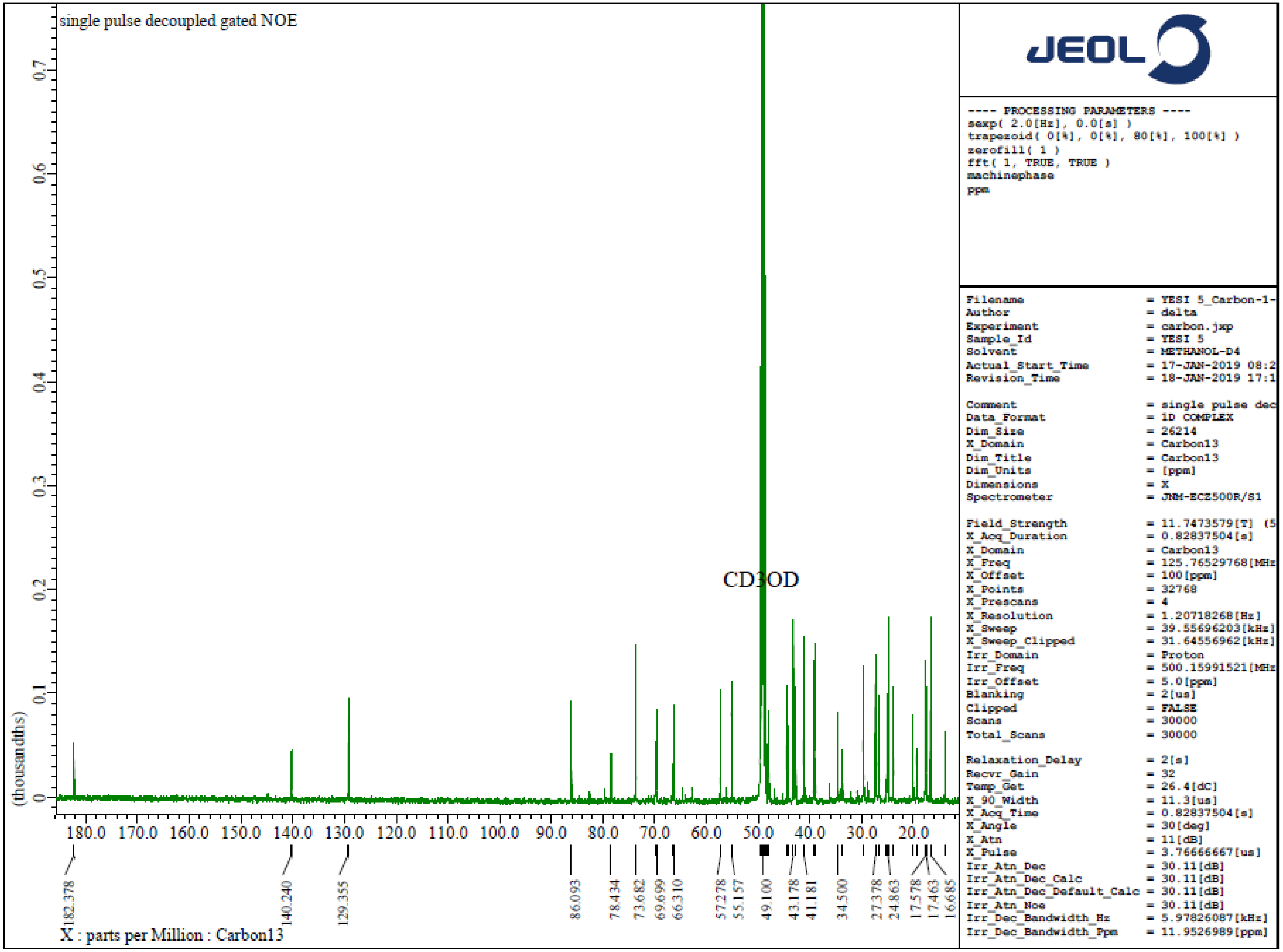


```
---- PROCESSING PARAMETERS ----
sexp( 2.0[Hz], 0.0[s] )
trapezoid( 0[%], 0[%], 80[%], 100[%] )
zerofill( 1 )
fft( 1, TRUE, TRUE )
machinephase
ppm
```

```
Filename                  = YESI 5_Carbon-1-
Author                    = delta
Experiment                = carbon.jxp
Sample_Id                 = YESI 5
Solvent                   = METHANOL-D4
Actual_Start_Time         = 17-JAN-2019 08:2
Revision_Time             = 18-JAN-2019 17:1

Comment                   = single pulse dec
Data_Format               = 1D COMPLEX
Dim_Size                  = 26214
X_Domain                  = Carbon13
Dim_Title                 = Carbon13
Dim_Units                 = [ppm]
Dimensions                = X
Spectrometer              = JNM-ECZ500R/S1

Field_Strength            = 11.7473579[T] (5
X_Acq_Duration            = 0.82837504[s]
X_Domain                  = Carbon13
X_Freq                    = 125.76529768[MHz
X_Offset                  = 100[ppm]
X_Points                  = 32768
X_Prescans                = 4
X_Resolution              = 1.20718268[Hz]
X_Sweep                   = 39.55696203[kHz]
X_Sweep_Clipped           = 31.64556962[kHz]
Irr_Domain                = Proton
Irr_Freq                  = 500.15991521[MHz
Irr_Offset                = 5.0[ppm]
Blanking                  = 2[us]
Clipped                   = FALSE
Scans                     = 30000
Total_Scans               = 30000

Relaxation_Delay          = 2[s]
Recvr_Gain                = 32
Temp_Get                  = 26.4[dC]
X_90_Width                = 11.3[us]
X_Acq_Time                = 0.82837504[s]
X_Angle                   = 30[deg]
X_Atn                     = 11[dB]
X_Pulse                   = 3.76666667[us]
Irr_Atn_Dec               = 30.11[dB]
Irr_Atn_Dec_Calc          = 30.11[dB]
Irr_Atn_Dec_Default_Calc  = 30.11[dB]
Irr_Atn_Noe               = 30.11[dB]
Irr_Dec_Bandwidth_Hz      = 5.97826087[kHz]
Irr_Dec_Bandwidth_Ppm     = 11.9526989[ppm]
```

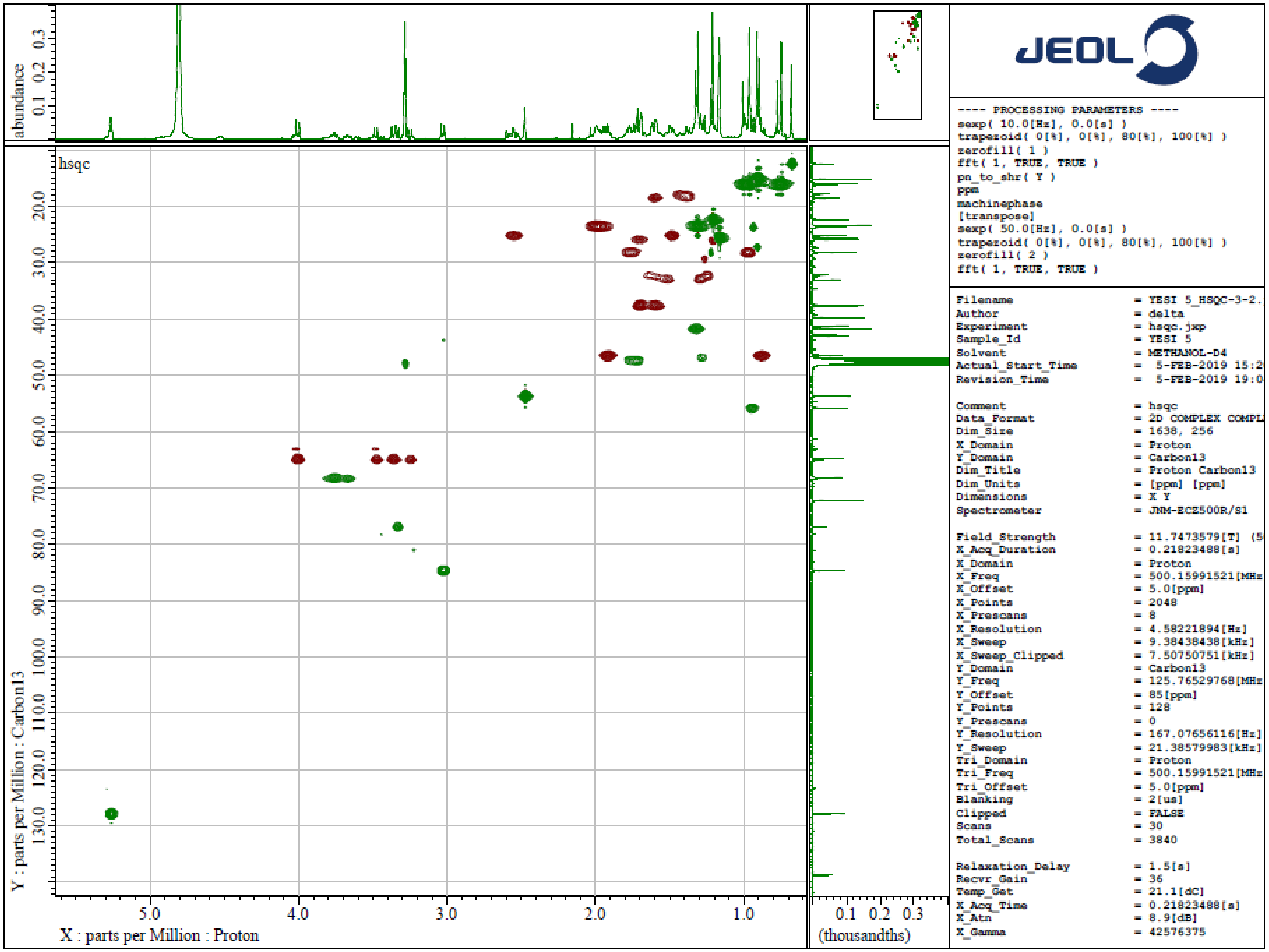

---- PROCESSING PARAMETERS ----

```
sexp( 10.0[Hz], 0.0[s] )
trapezoid( 0[%], 0[%], 80[%], 100[%] )
zerofill( 1 )
fft( 1, TRUE, TRUE )
pn_to_shr( Y )
ppm
machinephase
[transpose]
sexp( 50.0[Hz], 0.0[s] )
trapezoid( 0[%], 0[%], 80[%], 100[%] )
zerofill( 2 )
fft( 1, TRUE, TRUE )
```

Filename	= YESI 5_HSQC-3-2.
Author	= delta
Experiment	= hsqc.jxp
Sample_Id	= YESI 5
Solvent	= METHANOL-D4
Actual_Start_Time	= 5-FEB-2019 15:2
Revision_Time	= 5-FEB-2019 19:0
Comment	= hsqc
Data_Format	= 2D COMPLEX COMPL
Dim_Size	= 1638, 256
X_Domain	= Proton
Y_Domain	= Carbon13
Dim_Title	= Proton Carbon13
Dim_Units	= [ppm] [ppm]
Dimensions	= X Y
Spectrometer	= JNM-ECZ500R/S1
Field_Strength	= 11.7473579[T] (5
X_Acq_Duration	= 0.21823488[s]
X_Domain	= Proton
X_Freq	= 500.15991521[MHz
X_Offset	= 5.0[ppm]
X_Points	= 2048
X_Prescans	= 8
X_Resolution	= 4.58221894[Hz]
X_Sweep	= 9.38438438[kHz]
X_Sweep_Clipped	= 7.50750751[kHz]
Y_Domain	= Carbon13
Y_Freq	= 125.76529768[MHz
Y_Offset	= 85[ppm]
Y_Points	= 128
Y_Prescans	= 0
Y_Resolution	= 167.07656116[Hz]
Y_Sweep	= 21.38579983[kHz]
Tri_Domain	= Proton
Tri_Freq	= 500.15991521[MHz
Tri_Offset	= 5.0[ppm]
Blanking	= 2[us]
Clipped	= FALSE
Scans	= 30
Total_Scans	= 3840
Relaxation_Delay	= 1.5[s]
Recvr_Gain	= 36
Temp_Get	= 21.1[dC]
X_Acq_Time	= 0.21823488[s]
X_Atn	= 8.9[dB]
X_Gamma	= 42576375

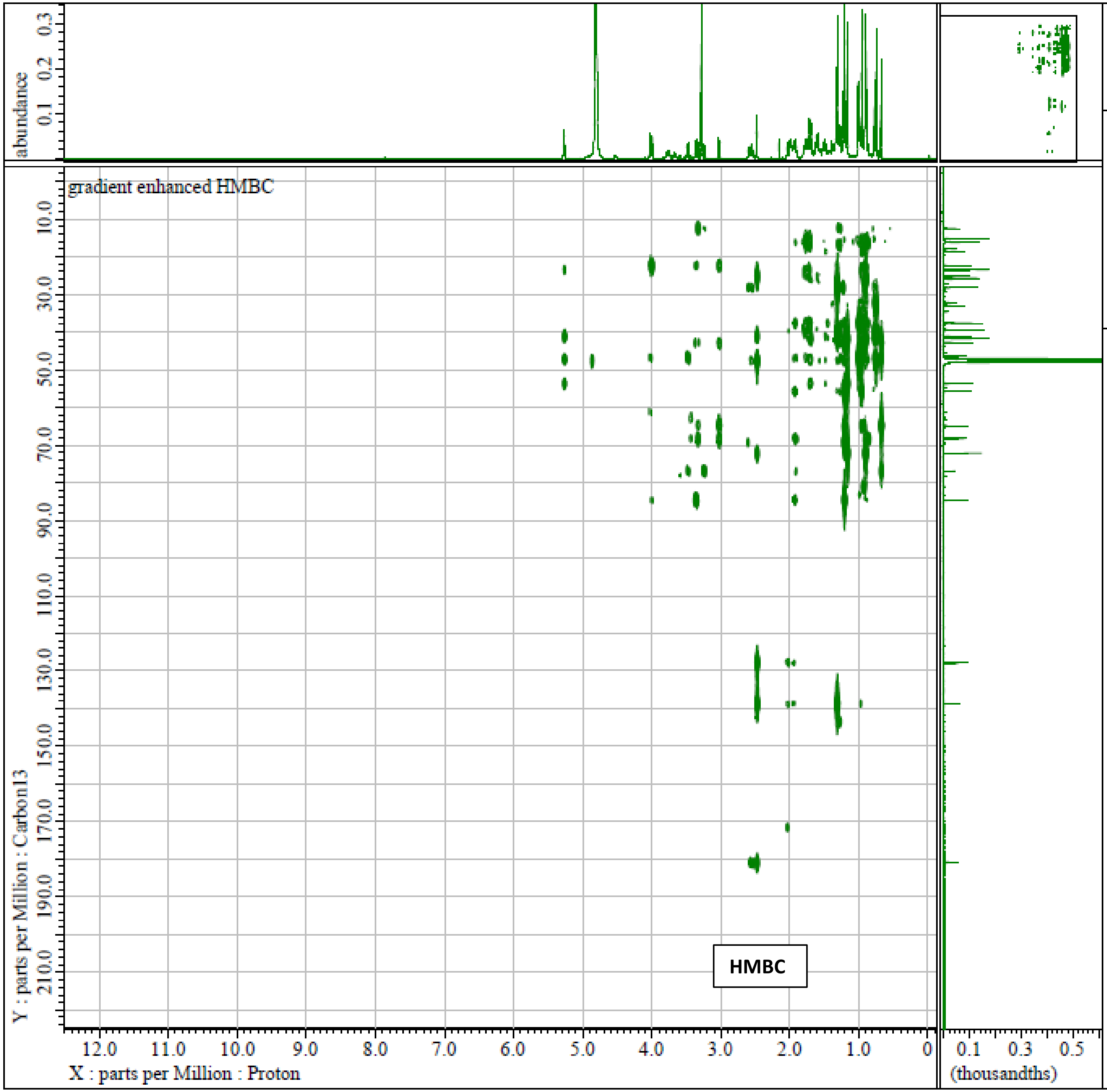


```
---- PROCESSING PARAMETERS ----
sexp( 0.2[Hz], 0.0[s] )
trapezoid( 0[%], 0[%], 80[%], 100[%] )
zerofill( 1 )
fft( 1, TRUE, TRUE )
machinephase
ppm

Filename             = YESI 5_HMBC-2-2.jdf
Author               = delta
Experiment           = hmbc.jxp
Sample_Id            = YESI 5
Solvent              = METHANOL-D4
Actual_Start_Time    =  5-FEB-2019 08:19:4
Revision_Time        =  5-FEB-2019 12:51:4

Comment              = gradient enhanced H
Data_Format          = 2D REAL REAL
Dim_Size             = 3277, 512
X_Domain             = Proton
Y_Domain             = Carbon13
Dim_Title            = Proton Carbon13
Dim_Units            = [ppm] [ppm]
Dimensions           = X Y
Spectrometer         = JNM-ECZ500R/S1

Field_Strength       = 11.7473579[T] (500[
X_Acq_Duration       = 0.43646976[s]
X_Domain             = Proton
X_Freq               = 500.15991521[MHz]
X_Offset             = 5.0[ppm]
X_Points             = 4096
X_Prescans           = 4
X_Resolution         = 2.29110947[Hz]
X_Sweep              = 9.38438438[kHz]
X_Sweep_Clipped      = 7.50750751[kHz]
Y_Domain             = Carbon13
Y_Freq               = 125.76529768[MHz]
Y_Offset             = 100[ppm]
Y_Points             = 256
Y_Prescans           = 0
Y_Resolution         = 122.83805031[Hz]
Y_Sweep              = 31.44654088[kHz]
Tri_Domain           = Proton
Tri_Freq             = 500.15991521[MHz]
Tri_Offset           = 5.0[ppm]
Blanking             = 2[us]
Clipped              = FALSE
Scans                = 32
Total_Scans          = 8192

Relaxation_Delay     = 1.5[s]
Recvr_Gain           = 36
Temp_Get             = 21[dC]
X_Acq_Time           = 0.43646976[s]
X_Atn                = 8.9[dB]
X_Gamma              = 42576375
```

Created by: Administrator, UNIFI

Created on: Jul 18, 2019

Item name: Process_Data_Yesi_FFUI

Created time: 09:42:00 SE Asia Standard Time

Item name: DFM

Item description:

Channel name: High energy : Time 7.9309 +/- 0.0340 minutes

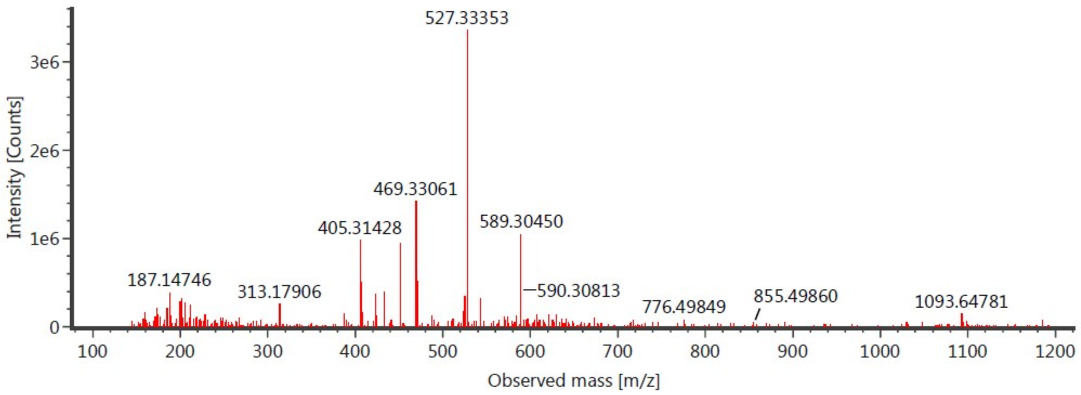

Supplement: Supplementary file 1 — Supplementary Information. [file 41598_2021_99970_MOESM1_ESM.pdf]
